# Serum Branched-Chain Amino Acids and Chronic Rheumatic Heart Diseases: Evidence From a Population-Based Prospective Study

**DOI:** 10.31083/RCM44157

**Published:** 2025-12-23

**Authors:** Lingling Xu, Zhixing Fan, Bo Pang

**Affiliations:** ^1^Department of Hematology and Rheumatology, The Sixth Hospital of Wuhan, Affiliated Hospital of Jianghan University, 430022 Wuhan, Hubei, China; ^2^Department of Cardiology, The First College of Clinical Medical Sciences, China Three Gorges University, 443003 Yichang, Hubei, China

**Keywords:** branched-chain amino acids, chronic rheumatic heart diseases, immune markers, therapeutic targets

## Abstract

**Background:**

This study aimed to systematically investigate the association between serum branched-chain amino acids (BCAAs) and the risk of chronic rheumatic heart disease (RHD), as well as to explore potential mediating mechanisms through immune markers.

**Methods:**

The data utilized in this prospective cohort study were derived from the UK Biobank. Serum BCAAs (leucine, isoleucine, and valine) were measured using metabolic profiling of nuclear magnetic resonance data. Chronic RHD cases were identified through hospital inpatient records and death registries. Multivariable Cox regression models were used to analyze the association between BCAAs and RHD risk. Causal mediation analysis was employed to investigate the mediated role of immune markers.

**Results:**

A total of 273,595 participants were included, with 6051 (2.21%) participants developing chronic RHD. Each one-unit standard deviation increase in total BCAAs was associated with a 4.8% increased risk of RHD (hazard ratio (HR) = 1.048, 95% confidence interval (CI): 1.023–1.074). Among individual BCAAs, valine exhibited the strongest association (HR = 1.061, 95% CI: 1.035–1.088). Subgroup analyses revealed significantly stronger associations in participants aged <65 years compared to those aged ≥65 years (*p* for interaction = 0.032). Mediation analysis demonstrated that immune markers significantly mediated the BCAA–RHD association, with lymphocyte-to-C-reactive protein ratio accounting for 30.8% of the total effect.

**Conclusions:**

Observational data suggest serum BCAAs correlate with increased RHD risk, especially in individuals aged <65 years; however, causation requires experimental verification. Immune markers significantly mediate the BCAA–RHD association, indicating that immunomodulatory pathways may be potential therapeutic targets. These findings provide novel insights into RHD pathogenesis and may inform risk stratification and prevention strategies.

## 1. Introduction

Rheumatic heart disease (RHD) represents one of the most significant yet preventable cardiovascular conditions globally, continuing to impose a substantial burden on public health systems worldwide [1, 2]. Despite being eminently preventable for over 70 years, RHD is the predominant cause of cardiovascular morbidity and early death among young people worldwide, with a particular impact on those in low- and middle-income countries [3, 4]. Current epidemiological data reveal alarming statistics: an estimated 54.8 million cases of RHD existed globally in 2021, with South Asia alone accounting for 57.6% of global RHD-related deaths [3, 5]. The disease demonstrates particularly high prevalence rates in endemic regions, with African populations showing rates of 18.41 per 1000 individuals [6]. This substantial disease burden translates into significant economic costs and prolonged disability, disproportionately affecting children and young adults in their most energetic years [7]. RHD primarily results from an autoimmune response triggered by group A β-hemolytic streptococcal infections, wherein molecular mimicry between streptococcal antigens and cardiac tissues initiates a cascade of inflammatory processes targeting heart valves [8]. Given this immunological pathogenesis, investigating susceptibility factors that influence immune system function and predispose individuals to RHD development represents a critical research priority for understanding disease mechanisms and identifying potential therapeutic targets [9, 10].

Branched-chain amino acids (BCAAs), which include leucine, isoleucine, and valine, are essential amino acids that serve fundamental roles in protein homeostasis, energy balance, and cellular signaling pathways [11, 12]. These amino acids function as critical building blocks for protein synthesis while simultaneously acting as signaling molecules that activate diverse biological programs ranging from protein synthesis to mitochondrial biogenesis [12, 13]. Accumulating evidence from recent research has established compelling associations between elevated serum BCAA levels and increased risk of various cardiovascular diseases, including obesity, hypertension, type 2 diabetes, heart failure, and atherosclerotic cardiovascular disease [11, 14]. Clinical studies have consistently demonstrated that disrupted BCAA homeostasis contributes to the pathophysiology of cardiometabolic diseases through tissue-specific and disease-specific regulatory mechanisms [13, 15]. However, despite the well-established connections between BCAAs and cardiovascular pathology, the relationship between serum BCAAs and chronic RHD remains virtually unexplored, with only limited preliminary evidence suggesting potential protective effects of modest leucine supplementation in RHD prevention [16]. Importantly, emerging research has revealed that BCAAs possess significant immunomodulatory properties [17, 18]. Nevertheless, whether BCAAs influence RHD development through immunomodulatory mechanisms remains to be elucidated, representing a critical knowledge gap that warrants systematic investigation.

This study, informed by a large-scale prospective cohort from the UK Biobank, aims to systematically evaluate the impact of serum BCAA levels on RHD risk and investigate the potential mediating role of immune markers. The findings will deliver a theoretical base and innovative research directions for future endeavors in the prevention, risk stratification, and treatment protocols of RHD.

## 2. Methods

### 2.1 Study Design

Utilizing data from the UK Biobank, a large-scale population-based biomedical database, this prospective cohort study included approximately 502,132 participants aged 40–69 years, recruited from 22 assessment centers throughout England, Scotland, and Wales during the period of 2006–2010. Participants underwent comprehensive baseline assessments including touchscreen questionnaires, physical examinations, and biological sample collection. The detailed study protocols and methodologies, previously published, can be accessed publicly via the UK Biobank website (https://www.ukbiobank.ac.uk). The North-West Multi-centre Research Ethics Committee (reference 16/NW/0274) approved the study, and all participants provided written informed consent in line with the Declaration of Helsinki principles.

Participants were excluded if they: (i) lacked measurement of BCAAs at baseline (n = 227,918), (ii) had incomplete BCAAs data (n = 213), (iii) had a history of RHD at baseline assessment (n = 406). Following these exclusions, our final analytical cohort comprised 273,595 participants who met all inclusion criteria and had complete baseline BCAA measurements available for analysis (**Supplementary Fig. 1**).

### 2.2 Assessment of Serum BCAAs

Nightingale Health Ltd. (Helsinki, Finland) developed the high-throughput NMR-based metabolic profiling platform used for measuring serum BCAAs concentrations (https://biobank.ndph.ox.ac.uk/showcase/label.cgi?id=220). This study analyzed isoleucine, leucine, and valine as the specific BCAAs, with the total BCAAs concentration calculated from the sum of these three amino acids. Individual BCAAs levels and total BCAA concentrations were then categorized into quartile (0.108 < Q1 ≤ 0.306 mmol/L; 0.306 < Q2 ≤ 0.353 mmol/L; 0.353 < Q3 ≤ 0.411 mmol/L; 0.411 < Q4 ≤ 1.305 mmol/L).

### 2.3 Ascertainment of Chronic Rheumatic Heart Diseases

The outcome was the incidence of chronic RHD. The International Classification of Diseases (ICD-9 and ICD-10) coding systems were used to identify RHD cases through hospital inpatient records (primary or secondary hospital diagnosis) and death registry records (underlying or contributory cause of death) (**Supplementary Table 1**). Chronic RHD defined by ≥2 hospitalizations with ICD-9 or ICD-10 code, or RHD as primary cause of death in registries. Participants were followed up starting from their enrollment date. The follow-up ended when any of these events first occurred: the participant developed RHD, the participant passed away, or the study reached its cutoff date.

### 2.4 Immunity Markers

Four established inflammatory and immune-related biomarkers were calculated as composite indices to assess systemic immune status and inflammatory burden. The albumin-to-alkaline phosphatase ratio (AAPR) was determined by dividing the level of serum albumin by that of alkaline phosphatase. The lymphocyte-to-monocyte ratio (LMR) was calculated through division of the absolute lymphocyte count by the absolute monocyte count. Meanwhile, the lymphocyte-to-C-reactive protein ratio (LCR) was obtained via division of lymphocyte count by C-reactive protein concentration. The platelet-to-lymphocyte ratio (PLR) was figured out through the division of the platelet count by the lymphocyte count.

### 2.5 Covariates Measurement

Demographic variables included participant age (categorized as <65 years and ≥65 years), sex (male and female), and race (Other and White). Socioeconomic indicators comprised educational attainment (university degree vs. no university degree) and annual household income (<£18,000 vs. >£18,000). Anthropometric measurements encompassed body mass index (BMI), which was calculated based on height and weight. Lifestyle factors encompassed physical activity (low/moderate/high), smoking history (never/previous/current), and alcohol consumption patterns (never/previous/current). The quality of the diet was evaluated by the Dietary Approaches to Stop Hypertension (DASH) score, which came from the responses to a food frequency questionnaire. Medical history was ascertained through participant self-reports combined with linked health records, including previous diagnoses of hypertension, diabetes mellitus, cardiovascular disease, and cancer. Cardiovascular disease history included atrial fibrillation, ischemic heart disease, heart failure, and stroke.

### 2.6 Statistical Analyses

For covariates with missing data, multiple imputation by chained equations was applied, performing five imputations. The predictor variables used in the imputation model included age, sex, race, education, income, physical activity, smoking, alcohol consumption, DASH score, BMI and history of hypertension, diabetes mellitus, cardiovascular disease (CVD), and cancer. The proportion of missing covariates was all within 30% (**Supplementary Table 2**), and they were missing randomly (**Supplementary Fig. 2**). Continuous variables were reported as means alongside standard deviations (SD) or as medians with interquartile ranges. Categorical variables, on the other hand, were shown as counts and percentages. For normally distributed variables, group comparisons were conducted using ANOVA or Student’s *t*-test. For non-normally distributed variables, the Kruskal-Wallis rank sum test or Mann-Whitney U test was applied. Categorical variables were assessed using the chi-squared test or Fisher’s exact test as appropriate.

To explore the connection between BCAAs and RHD risk, we utilized multivariable Cox proportional hazard models to derive hazard ratios (HRs) and 95% confidence intervals (CIs). The BCAAs data underwent transformation into z-scores, with HRs assessed for each SD unit rise. Additionally, BCAAs were stratified into quartiles to scrutinize the influence of quartile increments on RHD risk and to conduct trend tests. Four progressive models were established: crude model (unadjusted), Model 1 adjusted for sociodemographic characteristics (age, sex, race, education, and income); Model 2 further adjusted for life-style (physical activity, smoking, alcohol consumption, and DASH score); and Model 3 additionally adjusted for disease and health status (BMI and history of hypertension, diabetes mellitus, CVD, and cancer). To investigate the dose-response relationship between BCAA levels and RHD events, restricted cubic splines (RCS) were employed. The RCS analysis was fully adjusted for all covariates included in Model 3.

Using the “CMAverse” R package, causal mediation analysis was performed to explore how immune markers (AAPR, LMR, LCR, and PLR) might mediate the relationship between BCAAs and RHD risk. This approach employs counterfactual-based mediation analysis, which allows for the dissecting the total effect into natural direct and indirect effects within the counterfactual framework [19]. Bootstrap resampling (n = 100) was used to calculate 95% CI for the mediation effects. The mediated proportion was determined by dividing the natural indirect effect by the total effect, representing the percentage of the total association that operates through each immune marker.

To evaluate potential disparities in study outcomes among different population groups, stratification was performed based on age (<65 vs. ≥65 years), sex, education, race, income, physical activity, smoking status, and alcohol consumption. Interaction terms were tested to evaluate effect modification. For the subgroups with a small sample size, we conducted a post hoc power analysis using the powerSurvEpi R software package (version 0.1.5, Vienna, Austria). We performed several sensitivity analyses to test the robustness of our study findings. Firstly, the accelerated failure time model with Weibull distribution was employed as an alternative analytical approach. Secondly, we conducted Cox regression models using age as the time scale. Thirdly, to evaluate the influence of missing data imputation, participants with incomplete baseline covariate data were excluded. Fourthly, to reduce the reverse causality effect, participants who developed RHD within the first 2 years of follow-up were excluded. Finally, participants with a history of CVD (ischemic heart disease, heart failure, and stroke) were excluded to reduce potential confounding.

Statistical analyses were performed using R software (version 4.5.0, R Foundation, Vienna, Austria). The statistical significance of the differences between groups was determined by a two-tailed *p* value less than 0.05. In this study, we analyzed four exposure factors and four immune markers. The significance level was adjusted using the Bonferroni correction method: for the association between BCAAs and RHD, the significance level was set at 0.05/4 = 0.0125; for the mediation analysis, the significance level was set at 0.05/16 = 0.003.

## 3. Results

### 3.1 Baseline Characteristics

We included a total of 273,595 participants in the final analysis. The distribution of covariates remains essentially consistent before and after imputation for missing data (**Supplementary Table 3**). Over a median follow-up period of 13.71 ± 1.17 years, chronic RHD occurred in 6051 (2.21%) participants. Baseline characteristics demonstrated distinct patterns across BCAA quartiles (Table 1). Participants in higher BCAA quartiles were more likely to be male (25.90% in Q1 vs. 61.13% in Q4), had higher BMI (25.76 ± 4.40 kg/m^2^ in Q1 vs. 28.89 ± 4.87 kg/m^2^ in Q4), and showed increased prevalence of diabetes mellitus (2.67% vs. 9.71%), hypertension (25.20% vs. 36.73%), and CVD (6.17% vs. 10.24%). Conversely, DASH scores decreased progressively across quartiles (5.01 ± 1.34 in Q1 vs. 4.80 ± 1.34 in Q4).

**Table 1.** **Baseline characteristics stratified by total BCAA quartile levels**.

Characteristic	Level	Overall	Q1	Q2	Q3	Q4	*p*
		(n = 273,595)	(n = 68,411)	(n = 68,391)	(n = 68,404)	(n = 68,389)	
Age (mean (SD))	Continuous variable (years)	56.56 (8.08)	56.08 (8.29)	56.77 (8.06)	56.76 (8.00)	56.64 (7.96)	<0.001
	<65 years	221,287 (80.88)	55,693 (81.41)	54,820 (80.16)	55,088 (80.53)	55,686 (81.43)	<0.001
	≥65 years	52,308 (19.12)	12,718 (18.59)	13,571 (19.84)	13,316 (19.47)	12,703 (18.57)	
Sex (%)	Female	147,678 (53.98)	50,694 (74.10)	39,423 (57.64)	30,980 (45.29)	26,581 (38.87)	<0.001
	Male	125,917 (46.02)	17,717 (25.90)	28,968 (42.36)	37,424 (54.71)	41,808 (61.13)	
Race (%)	Other	13,584 (4.97)	2906 (4.25)	3241 (4.74)	3606 (5.27)	3831 (5.60)	<0.001
	White	260,011 (95.03)	65,505 (95.75)	65,150 (95.26)	64,798 (94.73)	64,558 (94.40)	
Education (%)	No university degree	186,114 (68.03)	46,476 (67.94)	46,902 (68.58)	46,551 (68.05)	46,185 (67.53)	0.001
	University degree	87,481 (31.97)	21,935 (32.06)	21,489 (31.42)	21,853 (31.95)	22,204 (32.47)	
Income (%)	<£18,000	65,492 (23.94)	17,476 (25.55)	16,675 (24.38)	15,886 (23.22)	15,455 (22.60)	<0.001
	>£18,000	208,103 (76.06)	50,935 (74.45)	51,716 (75.62)	52,518 (76.78)	52,934 (77.40)	
BMI (mean (SD))	Continuous variable (kg/m^2^)	27.46 (4.78)	25.76 (4.40)	27.12 (4.59)	28.06 (4.69)	28.89 (4.87)	<0.001
Physical activity (%)	Low	51,353 (18.77)	11,574 (16.92)	12,478 (18.25)	13,118 (19.18)	14,183 (20.74)	<0.001
	Moderate	109,618 (40.07)	27,621 (40.38)	27,380 (40.03)	27,391 (40.04)	27,226 (39.81)	
	High	112,624 (41.16)	29,216 (42.71)	28,533 (41.72)	27,895 (40.78)	26,980 (39.45)	
Smoke (%)	Never	149,478 (54.63)	38,617 (56.45)	37,693 (55.11)	36,868 (53.90)	36,300 (53.08)	<0.001
	Previous	95,173 (34.79)	21,866 (31.96)	23,446 (34.28)	24,548 (35.89)	25,313 (37.01)	
	Current	28,944 (10.58)	7928 (11.59)	7252 (10.60)	6988 (10.22)	6776 (9.91)	
Alcohol (%)	Never	11,917 (4.36)	3012 (4.40)	2997 (4.38)	2943 (4.30)	2965 (4.34)	<0.001
	Previous	9780 (3.57)	2579 (3.77)	2323 (3.40)	2316 (3.39)	2562 (3.75)	
	Current	251,898 (92.07)	62,820 (91.83)	63,071 (92.22)	63,145 (92.31)	62,862 (91.92)	
DASH (mean (SD))	Continuous variable	4.90 (1.34)	5.01 (1.34)	4.93 (1.34)	4.86 (1.34)	4.80 (1.34)	<0.001
History of diabetes mellitus (%)	No	258,506 (94.48)	66,583 (97.33)	65,782 (96.19)	64,393 (94.14)	61,748 (90.29)	<0.001
	Yes	15,089 (5.52)	1828 (2.67)	2609 (3.81)	4011 (5.86)	6641 (9.71)	
History of hypertension (%)	No	188,037 (68.73)	51,170 (74.80)	48,090 (70.32)	45,509 (66.53)	43,268 (63.27)	<0.001
	Yes	85,558 (31.27)	17,241 (25.20)	20,301 (29.68)	22,895 (33.47)	25,121 (36.73)	
History of CVD (%)	No	251,163 (91.80)	64,188 (93.83)	63,245 (92.48)	62,342 (91.14)	61,388 (89.76)	<0.001
	Yes	22,432 (8.20)	4223 (6.17)	5146 (7.52)	6062 (8.86)	7001 (10.24)	
History of cancer (%)	No	248,655 (90.88)	61,715 (90.21)	61,942 (90.57)	62,402 (91.23)	62,596 (91.53)	<0.001
	Yes	24,940 (9.12)	6696 (9.79)	6449 (9.43)	6002 (8.77)	5793 (8.47)	
RHD (%)	No	267,544 (97.79)	67,020 (97.97)	66,890 (97.81)	66,891 (97.79)	66,743 (97.59)	<0.001
	Yes	6051 (2.21)	1391 (2.03)	1501 (2.19)	1513 (2.21)	1646 (2.41)	
Follow up (mean (SD))	Continuous variable (years)	13.71 (1.17)	13.69 (1.17)	13.69 (1.15)	13.70 (1.17)	13.75 (1.18)	<0.001

BCAA, branched-chain amino acid; DASH, dietary approaches to stop hypertension; BMI, body mass index; CVD, cardiovascular disease; RHD, rheumatic heart diseases; SD, standard deviation.

RHD participants were older (61.96 ± 6.18 vs. 56.44 ± 8.08 years), had higher BMI, and demonstrated greater comorbidity burden compared to controls (**Supplementary Table 4**). In the RHD group, total BCAA concentrations were significantly higher (*p* < 0.001), with similar patterns observed for individual components (Table 2). Immune markers revealed significancal difference between two groups. Specifically, RHD patients had notably lower AAPR (0.530 vs. 0.563), LMR (3.800 vs. 4.196), LCR (1.065 vs. 1.408), and PLR (128.743 vs. 132.632), with all *p*-values being less than 0.001.

**Table 2.** **BCAAs levels and immune characteristics in RHD and control group**.

Characteristic	Level	Overall	Control	RHD	*p*
		(n = 273,595)	(n = 267,544)	(n = 6051)	
BCAAs (median [IQR])		0.353 [0.306, 0.411]	0.353 [0.306, 0.411]	0.358 [0.310, 0.417]	<0.001
	Q1	68,411 (25.00)	67,020 (25.05)	1391 (22.99)	<0.001
	Q2	68,391 (25.00)	66,890 (25.00)	1501 (24.81)	
	Q3	68,404 (25.00)	66,891 (25.00)	1513 (25.00)	
	Q4	68,389 (25.00)	66,743 (24.95)	1646 (27.20)	
Leucine (median [IQR])		0.100 [0.085, 0.119]	0.100 [0.085, 0.119]	0.101 [0.085, 0.120]	0.032
	Q1	68,403 (25.00)	66,919 (25.01)	1484 (24.52)	0.212
	Q2	68,417 (25.01)	66,943 (25.02)	1474 (24.36)	
	Q3	68,389 (25.00)	66,874 (25.00)	1515 (25.04)	
	Q4	68,386 (25.00)	66,808 (24.97)	1578 (26.08)	
Isoleucine (median [IQR])		0.048 [0.039, 0.060]	0.048 [0.039, 0.060]	0.049 [0.040, 0.060]	<0.001
	Q1	68,401 (25.00)	66,989 (25.04)	1412 (23.33)	<0.001
	Q2	68,401 (25.00)	66,942 (25.02)	1459 (24.11)	
	Q3	68,400 (25.00)	66,805 (24.97)	1595 (26.36)	
	Q4	68,393 (25.00)	66,808 (24.97)	1585 (26.19)	
Valine (median [IQR])		0.205 [0.180, 0.235]	0.205 [0.180, 0.235]	0.208 [0.183, 0.239]	<0.001
	Q1	68,424 (25.01)	67,045 (25.06)	1379 (22.79)	<0.001
	Q2	68,393 (25.00)	66,925 (25.01)	1468 (24.26)	
	Q3	68,389 (25.00)	66,865 (24.99)	1524 (25.19)	
	Q4	68,389 (25.00)	66,709 (24.93)	1680 (27.76)	
AAPR (median [IQR])		0.563 [0.468, 0.675]	0.563 [0.469, 0.676]	0.530 [0.440, 0.632]	<0.001
LMR (median [IQR])		4.185 [3.250, 5.333]	4.196 [3.250, 5.343]	3.800 [2.900, 5.000]	<0.001
LCR (median [IQR])		1.400 [0.674, 2.815]	1.408 [0.679, 2.830]	1.065 [0.502, 2.176]	<0.001
PLR (median [IQR])		132.531 [105.714, 166.842]	132.632 [105.825, 166.879]	128.743 [101.352, 164.198]	<0.001

AAPR, albumin to alkaline phosphatase ratio; LMR, lymphocyte-to-monocyte ratio; LCR, lymphocyte and C-reactive protein Ratio; PLR, platelet-to-Lymphocyte Ratio.

### 3.2 Association Between BCAAs and the Risk of RHD 

**Fig. 1.**
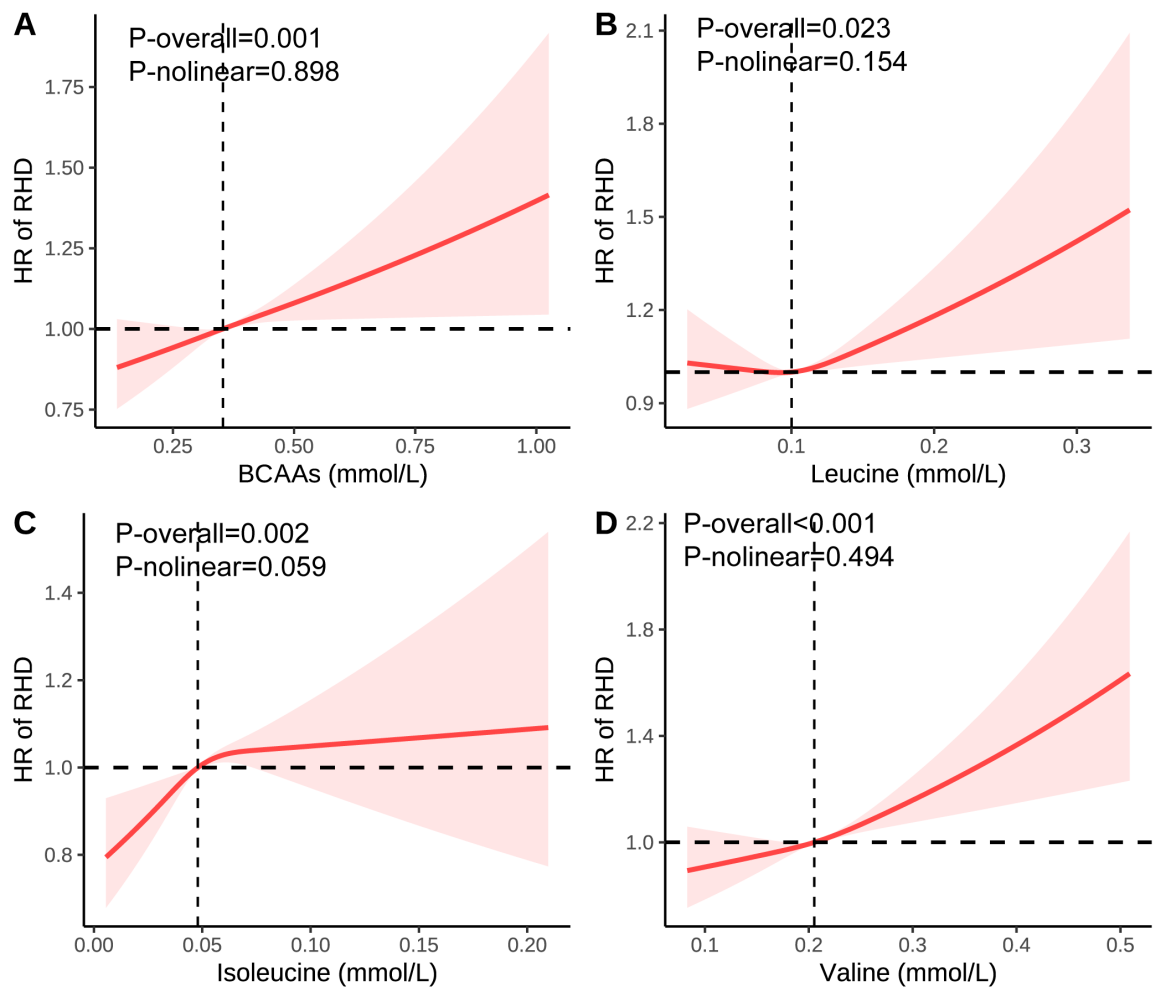
**Association of the BCAAs on RHD using RCS**. (A) Association of the BCAAs on RHD. (B) Association of the leucine on RHD. (C) Association of the isoleucine on RHD. (D) Association of the valine on RHD. Models were adjusted for age, sex, race, education, income, physical activity, smoke, alcohol, DASH, BMI, and the history of hypertension, diabetes mellitus, CVD, and cancer. BCAA, Branched-chain amino acid; DASH, Dietary approaches to stop hypertension; BMI, Body mass index; CVD, Cardiovascular disease; RHD, Rheumatic heart diseases; RCS, Restricted cubic spline; HR, Hazard ratio.

Serum BCAAs were significantly associated with RHD risk, as shown by multivariable Cox proportional hazard regression analyses (Table 3). We have verified the Cox proportional hazards assumption through Schoenfeld residual tests (**Supplementary Tables 5,6**; **Supplementary Figs. 3–11**). In Model 3, a 1-SD rise in total BCAAs corresponded to a 4.8% increased risk of RHD (HR = 1.048, 95% CI: 1.023–1.074, *p* < 0.001). Upon quartile analysis, individuals in the top quartile (Q4) exhibited an 8.3% higher risk than those in the bottom quartile (Q1) (HR = 1.083, 95% CI: 1.007–1.164, *p* = 0.032). HRs across BCAA quartiles, denoting the BCAAs concentration threshold (>0.411 mmol/L) for RHD risk. Among individual BCAAs, valine exhibited the strongest association with RHD risk, with each 1-SD increase conferring a 6.1% elevated risk (HR = 1.061, 95% CI: 1.035–1.088, *p* < 0.0001). Isoleucine showed a modest but significant association (HR = 1.039, 95% CI: 1.014–1.065, *p* = 0.002), while leucine demonstrated the weakest association (HR= 1.030, 95% CI: 1.004–1.056, *p* = 0.021). Restricted cubic spline analyses confirmed dose-response relationships between BCAAs and RHD risk (Fig. 1).

**Table 3.** **Association of BCAAs on the risk of chronic RHD using Cox regression**.

Exposure		Crude Model		Model 1		Model 2		Model 3	
		HR (95% CI)	*p*	HR (95% CI)	*p*	HR (95% CI)	*p*	HR (95% CI)	*p*
BCAAs per 1SD		1.049 (1.024, 1.075)	<0.001	1.055 (1.029, 1.081)	<0.0001	1.048 (1.022, 1.074)	<0.001	1.048 (1.023, 1.074)	<0.001
	Q1	ref		ref		ref		ref	
	Q2	1.080 (1.004, 1.161)	0.040	1.065 (0.990, 1.145)	0.092	1.058 (0.984, 1.139)	0.127	1.047 (0.973, 1.126)	0.218
	Q3	1.086 (1.010, 1.168)	0.026	1.082 (1.006, 1.164)	0.034	1.069 (0.994, 1.150)	0.073	1.035 (0.962, 1.113)	0.360
	Q4	1.174 (1.093, 1.261)	<0.0001	1.189 (1.107, 1.277)	<0.0001	1.165 (1.084, 1.251)	<0.0001	1.083 (1.007, 1.164)	0.032
*p* for trend			<0.0001		<0.0001		<0.0001		0.053
Leucine per 1SD		1.023 (0.998, 1.049)	0.067	1.036 (1.011, 1.062)	0.005	1.029 (1.004, 1.055)	0.024	1.030 (1.004, 1.056)	0.021
	Q1	ref		ref		ref		ref	
	Q2	0.994 (0.925, 1.068)	0.866	0.996 (0.927, 1.071)	0.917	0.989 (0.920, 1.063)	0.756	0.979 (0.911, 1.052)	0.566
	Q3	1.021 (0.951, 1.097)	0.566	1.040 (0.968, 1.117)	0.281	1.027 (0.956, 1.104)	0.460	1.000 (0.930, 1.074)	0.995
	Q4	1.055 (0.983, 1.133)	0.138	1.091 (1.017, 1.171)	0.016	1.069 (0.995, 1.148)	0.067	1.002 (0.933, 1.077)	0.950
*p* for trend			0.099		0.008		0.038		0.807
Isoleucine per 1SD		1.043 (1.018, 1.069)	<0.001	1.045 (1.020, 1.071)	<0.001	1.039 (1.013, 1.064)	0.003	1.039 (1.014, 1.065)	0.002
	Q1	ref		ref		ref		ref	
	Q2	1.036 (0.963, 1.115)	0.339	1.022 (0.950, 1.099)	0.565	1.015 (0.943, 1.092)	0.697	1.005 (0.934, 1.081)	0.904
	Q3	1.132 (1.054, 1.216)	<0.001	1.131 (1.053, 1.215)	<0.001	1.115 (1.038, 1.198)	0.003	1.085 (1.009, 1.165)	0.027
	Q4	1.117 (1.040, 1.200)	0.003	1.123 (1.045, 1.206)	0.002	1.101 (1.025, 1.183)	0.009	1.037 (0.964, 1.115)	0.327
*p* for trend			<0.001		<0.001		<0.001		0.117
Valine per 1SD		1.065 (1.040, 1.092)	<0.0001	1.068 (1.042, 1.095)	<0.0001	1.061 (1.035, 1.087)	<0.0001	1.061 (1.035, 1.088)	<0.0001
	Q1	ref		ref		ref		ref	
	Q2	1.065 (0.990, 1.146)	0.092	1.042 (0.969, 1.122)	0.267	1.039 (0.965, 1.118)	0.307	1.028 (0.955, 1.107)	0.458
	Q3	1.103 (1.026, 1.186)	0.008	1.081 (1.005, 1.163)	0.036	1.072 (0.996, 1.153)	0.063	1.037 (0.964, 1.115)	0.334
	Q4	1.209 (1.126, 1.299)	<0.0001	1.208 (1.125, 1.297)	<0.0001	1.186 (1.104, 1.274)	<0.0001	1.099 (1.022, 1.181)	0.011
*p* for trend			<0.0001		<0.0001		<0.0001		0.012

Model 1 adjusted for age, sex, race, education and income. Model 2 adjusted for Model 1+ physical activity, smoke, alcohol, and DASH. Model 3 adjusted for Model 2+ BMI, and the history of hypertension, diabetes mellitus, CVD, and cancer. BCAA quartiles: 0.108 < Q1 ≤ 0.306 mmol/L; 0.306 < Q2 ≤ 0.353 mmol/L; 0.353 < Q3 ≤ 0.411 mmol/L; 0.411 < Q4 ≤ 1.305 mmol/L. HR, Hazard ratio; CI, Confidence interval.

### 3.3 Subgroup Analysis for the Association of BCAAs on RHD Risk

**Fig. 2.**
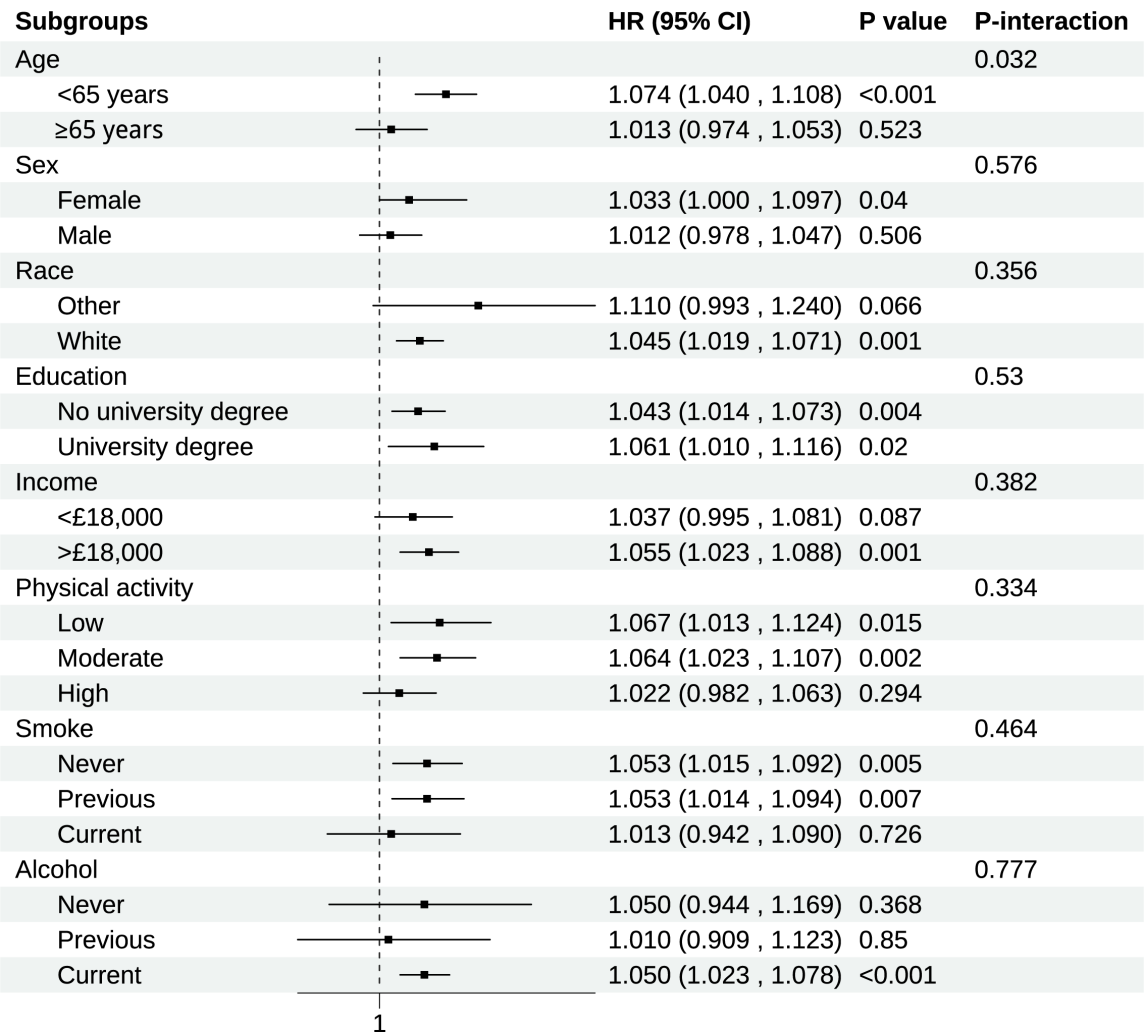
**Subgroup analysis of association of BCAAs on chronic RHD**. Models were adjusted for age, sex, race, education, income, physical activity, smoke, alcohol, DASH, BMI, and the history of hypertension, diabetes mellitus, CVD, and cancer. CI, Confidence interval.

**Fig. 3.**
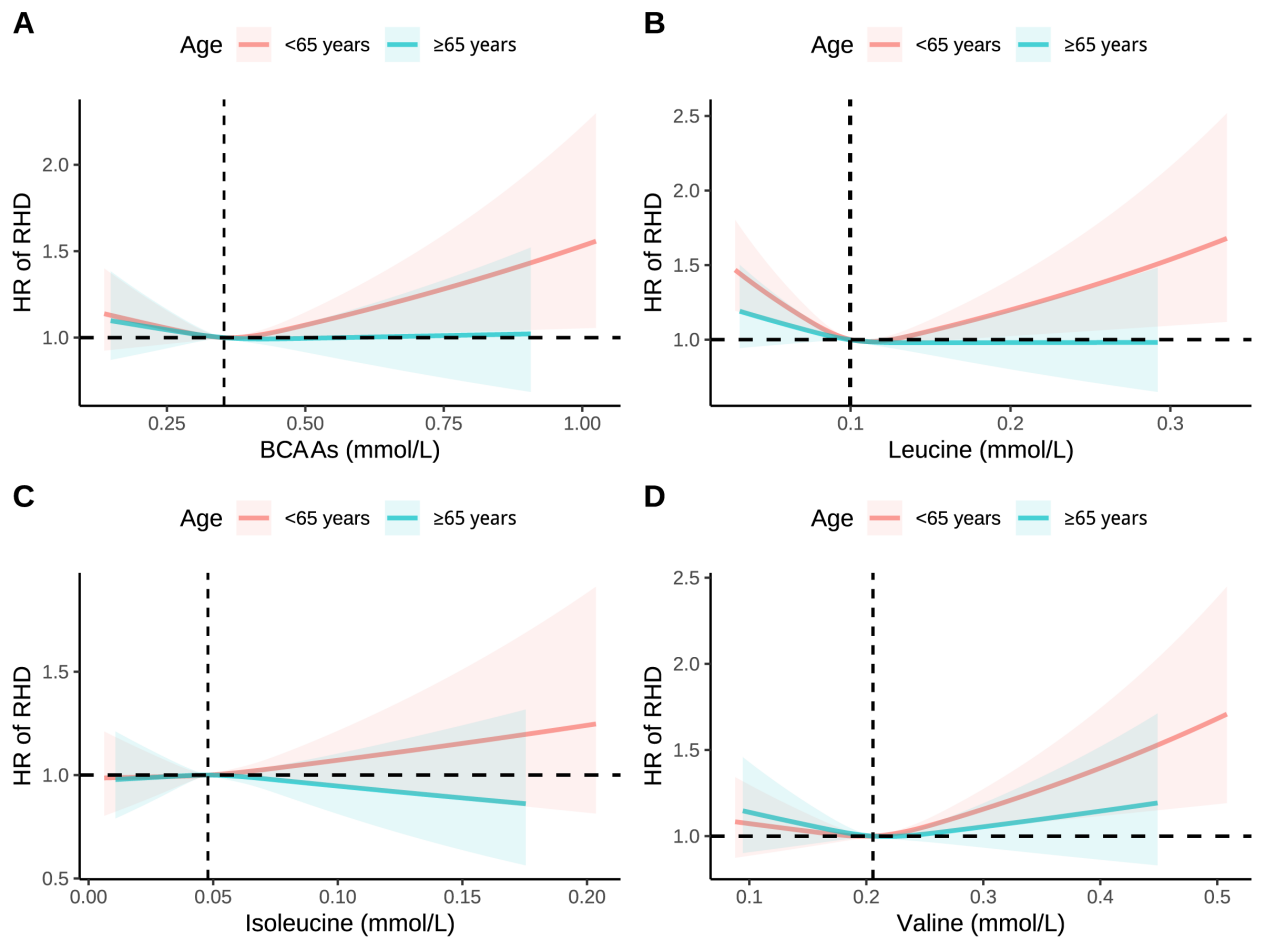
**RCS analysis of association between BCAAs with RHD stratified by age**. (A) Association between BCAAs with RHD stratified by age. (B) Association between leucine with RHD stratified by age. (C) Association between isoleucine with RHD stratified by age. (D) Association between valine with RHD stratified by age. Models were adjusted for age, sex, race, education, income, physical activity, smoke, alcohol, DASH, BMI, and the history of hypertension, diabetes mellitus, CVD, and cancer.

Consistent associations between BCAAs and RHD risk across different population strata were demonstrated in subgroup analyses (Fig. 2). The most notable finding was a significant age-related interaction (*p* for interaction = 0.032). Compared to older participants (≥65 years: HR = 1.013, 95% CI: 0.974–1.053, *p* = 0.523), younger participants exhibited stronger associations (<65 years: HR = 1.074, 95% CI: 1.040–1.108, *p* < 0.001). Significant interactions were not found for sex, education, income, or lifestyle factors including physical activity, smoking, and alcohol consumption. Individual BCAA analyses revealed consistent age-related patterns (**Supplementary Figs. 12–14**), with leucine, isoleucine, and valine all demonstrating stronger associations in younger participants. Age-stratified restricted cubic spline analyses further confirmed these differential associations (Fig. 3). In participants <65 years, all BCAAs exhibited progressive dose-response relationships with steeper risk curves, particularly for total BCAAs and valine.

In the subgroups with relatively small sample sizes, specifically “other” in race, “Current” in smoking status, and “Never” and “Previous” in alcohol consumption, no statistically significant associations were observed between BCAAs and RHD. To further elucidate this, we conducted post hoc power analyses for Cox regression with survival data using the powerSurvEpi R package. The statistical power to detect significant effects in these four subgroups was 2.5%, 2.6%, 4.2%, and 5.5%, respectively. These low power values suggest that the absence of statistical significance in these subgroups is likely attributable to insufficient statistical power, rather than a true absence of association.

### 3.4 Mediating Role of Immunity in the Association of BCAAs on RHD Risk

**Fig. 4.**
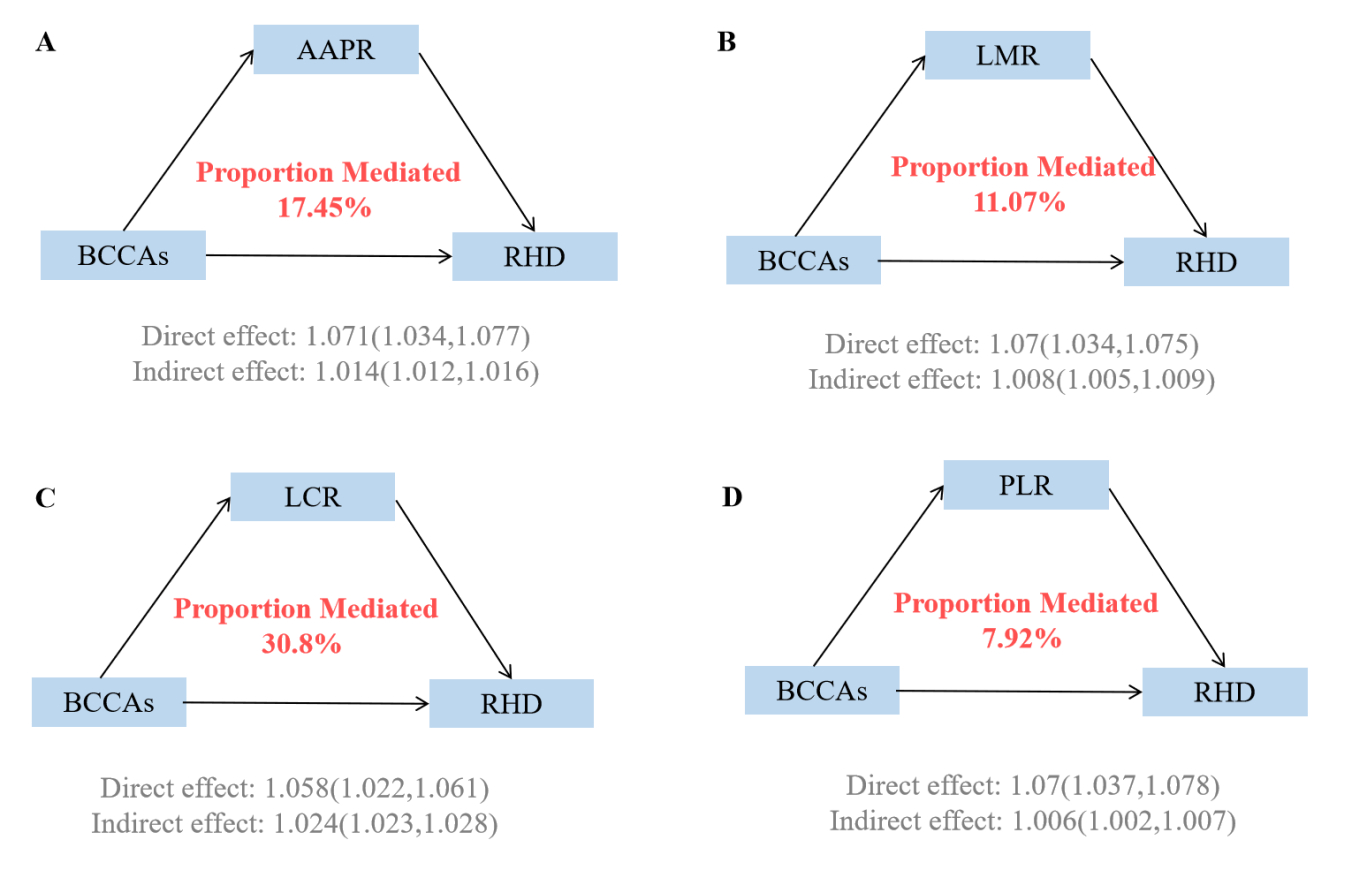
**Mediating proportion of immunity in the relationship between BCAAs and RHD**. (A) Mediating proportion of AAPR in the relationship between BCAAs and RHD. (B) Mediating proportion of LMR in the relationship between BCAAs and RHD. (C) Mediating proportion of LCR in the relationship between BCAAs and RHD. (D) Mediating proportion of PLR in the relationship between BCAAs and RHD. Models were adjusted for age, sex, race, education, income, physical activity, smoke, alcohol, DASH, BMI, and the history of hypertension, diabetes mellitus, CVD, and cancer. AAPR, Albumin to alkaline phosphatase ratio; LMR, Lymphocyte-to-monocyte ratio; LCR, Lymphocyte and C-reactive protein Ratio; PLR, Platelet-to-Lymphocyte Ratio.

Causal mediation analyses revealed that immune markers significantly mediated the associations between BCAAs and RHD risk (**Supplementary Table 7**, Fig. 4). Among the four immune indicators examined, LCR demonstrated the strongest mediating effect, accounting for 30.8% (95% CI: 32.53–52.87%) of the total association between BCAAs and RHD risk. The AAPR served as the second most important mediator, explaining 17.45% (95% CI: 17.47–32.58%) of the relationship. LMR contributed 11.07% (95% CI: 7.99–17.68%) to the mediation, while PLR had the smallest mediating effect at 7.92% (95% CI: 4.89–12.12%). Individual BCAAs analyses demonstrated consistent patterns with varying magnitudes (**Supplementary Tables 8,9**, **Supplementary Figs. 15,16**).

### 3.5 Sensitivity Analysis of the Association of BCAAs on RHD Risk

Multiple sensitivity analyses confirmed the robustness of our primary findings. The accelerated failure time model yielded consistent results, with each 1-SD increase in total BCAAs correlating to a 2.4% increased risk of RHD (HR = 1.024, 95% CI: 1.011–1.037) (**Supplementary Table 10**). Cox regression using the time scale of age produced nearly identical results to our primary analysis (**Supplementary Table 11**), with total BCAAs maintaining significance (HR = 1.048, 95% CI: 1.023–1.074). After excluding individuals with missing baseline covariates, the associations remained significant though slightly attenuated, with total BCAAs showing a 4.2% increased risk per 1-SD rise (HR = 1.042, 95% CI: 1.008–1.077) (**Supplementary Table 12**). To address potential reverse causality, excluding individuals with incident RHD during the initial 2-year follow-up period, total BCAAs correlated with a 5.1% higher risk (HR = 1.051, 95% CI: 1.024–1.077) (**Supplementary Table 13**). Finally, after excluding participants with baseline CVD history, the link stayed significant for total BCAAs (HR = 1.027, 95% CI: 1.011–1.057) (**Supplementary Table 14**). Corresponding RCS analyses consistently demonstrated dose-response relationships across all sensitivity analyses (**Supplementary Figs. 17–20**).

## 4. Discussion 

In this extensive prospective cohort study utilizing the UK Biobank database, we provide a comprehensive evidence that elevated serum BCAAs correlate with a higher risk of chronic RHD. Among individual BCAAs, valine exhibited the strongest association with RHD risk, followed by isoleucine and leucine. Notably, subgroup analyses revealed a significant age-related interaction, with stronger associations observed in younger participants (<65 years) compared to older individuals (≥65 years). Furthermore, mediation analyses identified immune markers as significant mediators of this association, with the LCR accounting for 30.8% of the total effect.

Our findings demonstrate both important consistencies and distinctions with previous research investigating the relationship between BCAAs and CVD. Wang *et al*. [20] showed that elevated serum isoleucine levels correlated with a 10% higher risk of CVD, which aligns directionally with our finding of a 3.9% increased RHD risk per 1-SD increment in isoleucine. Similarly, Sun *et al*. [21] reported that the highest quintile of BCAAs demonstrated a 7–12% increased risk of major adverse cardiovascular events relative to the second quintile, further supporting the positive association pattern between BCAAs and CVD risk. Notably, Xu *et al*. [22] provided causal evidence through Mendelian randomization, demonstrating that genetically predicted elevated BCAAs levels causally increased risks of peripheral arterial disease and stroke. Our study is the first to systematically explore the link between BCAAs and chronic RHD, a specific CVD subtype. While Ye *et al*. [16] previously suggested that modest leucine supplementation might benefit RHD prevention, our large-scale cohort study reveals that elevated serum BCAA levels are actually associated with increased RHD risk. This seemingly contradictory finding may reflect differential biological effects between endogenous serum BCAA levels and exogenous supplementation.

The biological mechanisms underlying the association between elevated serum BCAAs and increased RHD risk appear to be multifaceted, involving complex interactions between immunomodulatory pathways, inflammatory cascades, and tissue remodeling processes. Emerging evidence suggests that BCAAs exert profound immunomodulatory effects that may predispose individuals to autoimmune-mediated cardiac pathology characteristic of RHD [17, 18]. BCAA accumulation may significantly alter immune cell function and polarization. Notably, Huang *et al*. [23] revealed that BCAAs supplementation induces pro-inflammatory macrophage polarization via the IFNGR1/JAK1/STAT1 signaling pathway, while Yao *et al*. [24] showed that the accumulation of BCAAs alters glucose metabolism of CD8^+^T cells, thereby augmenting their functional efficacy. Recent studies have suggested that BCAAs can modulate immune responses through the activation of the mTOR pathway, leading to T-cell activation [25]. This activation can subsequently increase C-reactive protein (CRP) levels, which may contribute to reduced LCR [26]. These findings are particularly relevant to RHD pathogenesis, as the disease fundamentally results from aberrant immune respons-es where molecular mimicry between streptococcal antigens and cardiac tissues triggers sustained inflammatory processes [27, 28]. The immunomodulatory properties of BCAAs may therefore amplify the autoimmune cascade that characterizes RHD development. Furthermore, BCAAs have been shown to promote chronic inflammation through multiple pathways, including enhanced generation of pro-inflammatory cytokines like IL-1β, TNF-α, and monocyte chemotactic protein-1 [23, 29]. This inflammatory milieu aligns with the pathophysiology of immune-mediated inflammatory diseases affecting cardiovascular structures, where persistent inflammation drives progressive tissue damage and functional impairment [30, 31]. Additionally, an emerging role for BCAA metabolism in fibrosis development [32], suggesting that dysregulated BCAA homeostasis may contribute to the progressive fibrotic remodeling and valvular dysfunction that define chronic RHD. The convergence of these immunomodulatory, inflammatory, and fibrotic mechanisms provides a compelling biological rationale for our observed association between elevated BCAAs and increased RHD risk.

Our findings have significant ramifications for clinical practice and public health initiatives aimed at preventing and managing chronic RHD. From a clinical perspective, serum BCAA levels may serve as novel biomarkers for RHD risk stratification, particularly in younger populations where the associations were most pronounced. The identification of elevated BCAAs as a risk factor could facilitate the development of personalized screening protocols, especially in endemic regions where RHD remains a leading cause of cardiovascular morbidity among young adults. Furthermore, our mediation analysis revealing that immune markers account for substantial proportions of the BCAA-RHD association provides mechanistic insights that could inform targeted therapeutic interventions. Given that Buch *et al*. [30] highlighted the under-recognition of cardiovascular involvement in immune-mediated inflammatory diseases and the potential of immune modulators in improving cardiovascular outcomes, our findings suggest that monitoring BCAA levels alongside immune markers could enhance early detection strategies. Regarding public health, these results emphasize the significance of addressing metabolic health as part of comprehensive RHD prevention programs. As Rwebembera and Beaton [33] noted the renewed global push to tackle the current burden of RHD, incorporating BCAA assessment into existing healthcare structures could strengthen integrated models for RHD screening and prevention, particularly in the low- or middle-income regions where the disease burden remains highest.

This study presents several notable strengths that distinguish it from previous cardiovascular research and contribute significantly to the understanding of RHD pathogenesis. First, this represents the largest prospective cohort investigation to date examining the link between serum BCAAs and the risk of chronic RHD. Second, the utilization of high-throughput NMR-based metabolic profiling ensures precise and standardized BCAA measurements, while the comprehensive covariate adjustment minimizes potential confounding effects. Third, the rigorous sensitivity analyses, including accelerated failure time models and exclusion of early events to address reverse causality, demonstrate the robustness of our findings across different analytical approaches.

Despite these strengths, several limitations warrant consideration when interpreting our findings. First, being an observational study, it precludes us from establishing causality between elevated BCAA levels and RHD development, as residual confounding from unmeasured factors cannot be entirely excluded. Future research should focus on experimental validation. Second, the UK Biobank predominantly comprises participants of European ancestry, which might restrict the applicability of our results to other ethnic groups. This limitation is particularly relevant in endemic regions such as sub-Saharan Africa, where the RHD burden is highest and validation in African cohorts is essential. Third, RHD diagnosis relied on hospital discharge codes and death registries, which may introduce misclassification bias and potentially underestimate disease incidence, especially for mild or asymptomatic cases. Fourth, BCAAs measurements were obtained at a single baseline timepoint, precluding assessment of temporal changes in BCAAs levels or their dynamic relationship with RHD risk progression. Fifth, while we adjusted for major dietary factors using the DASH score, detailed nutritional data including protein intake and dietary BCAA consumption were not available, potentially influencing the observed associations. Sixth, the evolving nature of RHD presentation and management over time may influence the current applicability of our results for modern clinical practice. Seventh, our study analyzed multiple related exposures (total BCAAs and individual amino acids) and multiple immune markers, so we applied the Bonferroni correction method to adjust significance levels. Although these method helps to mitigate the risk of false positives, readers should still interpret the results with caution, considering the inherent limitations associated with multiple testing. Finally, although we used imputation to handle randomly missing data and thus avoided the reduction of sample size, the random missing mechanism may still mask potential non-random missing factors, which may have some impact on the accuracy and generalizability of the research results.

## 5. Conclusions 

This extensive prospective cohort study offers robust evidence linking elevated serum BCAAs to a heightened risk of chronic RHD. Notably, these associations were more pronounced in younger participants (<65 years), suggesting age-dependent susceptibility patterns that warrant targeted prevention strategies. Our mediation analysis revealed that immune markers, providing novel mechanistic insights into how metabolic dysregulation may predispose individuals to autoimmune-mediated cardiac pathology through immunomodulatory pathways.

## Availability of Data and Materials

The datasets used and analysed during the current study available from the corresponding author on reasonable request.

## Supplementary Material

Supplementary material associated with this article can be found, in the online version, at https://doi.org/10.31083/RCM44157.
