# Supplementary material for: Serum Branched-Chain Amino Acids and Chronic Rheumatic Heart Diseases: Evidence From a Population-Based Prospective Study
Source: Rev Cardiovasc Med. 2025 Dec 23;26(12):44157. doi: 10.31083/RCM44157 (PMC12780991; doi:10.31083/RCM44157)
Supplement: Supplementary file 1 [file 2153-8174-26-12-44157-s1.doc]

**Circulating Branched-Chain Amino Acids and the risk of Chronic Rheumatic Heart Diseases: Evidence from a Population-Based Prospective Study**

Supplementary Table 1 Definition of RHD	2

Supplementary Table 2 Missing data proportions	3

Supplementary Table 3 Baseline characteristics before and after imputation	3

Supplementary Table 4 Baseline characteristics in RHD and control group	4

Supplementary Table 5 Cox proportional hazards hypothesis test of BCAAs (quartile grouping variables) and chronic RHD	6

Supplementary Table 6 Cox proportional hazards hypothesis test of BCAAs (continuous variable) and chronic RHD	7

Supplementary Table 7 Mediation of immunity indicators in association between BCAAs and RHD	8

Supplementary Table 8 Mediation of immunity indicators in association between Isoleucine and RHD	8

Supplementary Table 9 Mediation of immunity indicators in association between Valine and RHD	9

Supplementary Table 10 Sensitivity analysis of association between BCAAs and chronic RHD using accelerated failure time model	10

Supplementary Table 11 Sensitivity analysis of association between BCAAs and chronic RHD using Cox Regression with age scale	11

Supplementary Table 12 Sensitivity analysis of association between BCAAs and chronic RHD after excluding the participants with missing covariates	12

Supplementary Table 13 Sensitivity analysis of association between BCAAs and chronic RHD after excluding the participants who developed RHD within 2 years of follow-up.	13

Supplementary Table 14 Sensitivity analysis of association between BCAAs and chronic RHD after excluding the participants who has the history of CVD	15

Supplementary Fig. 1 Flowchart of this study	17

Supplementary Fig. 2 Missing data patterns	17

Supplementary Fig. 3 Cox proportional hazards hypothesis test of BCAAs (quartile grouping variable) and chronic RHD	18

Supplementary Fig. 4 Cox proportional hazards hypothesis test of Leucine (quartile grouping variable) and chronic RHD	19

Supplementary Fig. 5 Cox proportional hazards hypothesis test of Isoleucine (quartile grouping variable) and chronic RHD	20

Supplementary Fig. 6 Cox proportional hazards hypothesis test of Valine (quartile grouping variable) and chronic RHD	21

Supplementary Fig. 7 Cox proportional hazards hypothesis test of BCAAs (continuous variable) and chronic RHD	22

Supplementary Fig. 8 Cox proportional hazards hypothesis test of Leucine (continuous variable) and chronic RHD	23

Supplementary Fig. 9 Cox proportional hazards hypothesis test of Isoleucine (continuous variable) and chronic RHD	24

Supplementary Fig. 10 Cox proportional hazards hypothesis test of Valine (continuous variable) and chronic RHD	25

Supplementary Fig. 11 Residual plot of Cox proportional hazards model diagnostic	26

Supplementary Fig. 12 Subgroup analysis of association between Leucine and chronic RHD	27

Supplementary Fig. 13 Subgroup analysis of association between Isoleucine and chronic RHD	28

Supplementary Fig. 14 Subgroup analysis of association between Valine and chronic RHD	29

Supplementary Fig. 15 Mediating proportion of immunity in the relationship between Isoleucine and RHD	30

Supplementary Fig. 16 Mediating proportion of immunity in the relationship between Valine and RHD	31

Supplementary Fig. 17 RCS analysis of association between BCAAs with RHD using Cox Regression with age scale	32

Supplementary Fig. 18 RCS analysis of association between BCAAs with RHD after excluding the participants with missing covariates	33

Supplementary Fig. 19 RCS analysis of association between BCAAs with RHD after excluding the participants who developed RHD within 2 years of follow-up.	34

Supplementary Fig. 20 RCS analysis of association between BCAAs with RHD after excluding the participants who has the history of CVD.	35

**Supplementary** **Table** **1** Definition of RHD

| Field Name | Field ID | Code and Definition |
| --- | --- | --- |
| Diagnoses-ICD 9 | 41271 | 393 Chronic rheumatic heart disease  394 Diseases of mitral valve  3940 Mitral stenosis  3942 Mitral stenosis with insufficiency  3949 Other and unspecified diseases of mitral valve  395 Diseases of aortic valve  3951 Rheumatic aortic insufficiency  3959 Other and unspecified diseases of aortic valve  396 Diseases of mitral and aortic valves  3969 Diseases of mitral and aortic valves  398 Other rheumatic heart disease  3989 Other and unspecified rheumatic heart disease |
| Diagnoses-ICD10 | 41270 | I05 Rheumatic mitral valve diseases  I05.0 Mitral stenosis  I05.1 Rheumatic mitral insufficiency  I05.2 Mitral stenosis with insufficiency  I05.8 Other mitral valve diseases  I05.9 Mitral valve disease, unspecified  I06 Rheumatic aortic valve diseases  I06.0 Rheumatic aortic stenosis  I06.1 Rheumatic aortic insufficiency  I06.2 Rheumatic aortic stenosis with insufficiency  I06.8 Other rheumatic aortic valve diseases  I06.9 Rheumatic aortic valve disease, unspecified  I07 Rheumatic tricuspid valve diseases  I07.0 Tricuspid stenosis  I07.1 Tricuspid insufficiency  I07.2 Tricuspid stenosis with insufficiency  I07.8 Other tricuspid valve diseases  I07.9 Tricuspid valve disease, unspecified  I08 Multiple valve diseases  I08.0 Disorders of both mitral and aortic valves  I08.1 Disorders of both mitral and tricuspid valves  I08.2 Disorders of both aortic and tricuspid valves  I08.3 Combined disorders of mitral, aortic and tricuspid valves  I08.8 Other multiple valve diseases  I08.9 Multiple valve disease, unspecified  I09 Other rheumatic heart diseases  I09.0 Rheumatic myocarditis  I09.1 Rheumatic diseases of endocardium, valve unspecified  I09.8 Other specified rheumatic heart diseases  I09.9 Rheumatic heart disease, unspecified |
| Underlying (primary) cause of death: ICD10 | 40001 |  |
| Contributory (secondary) causes of death: ICD10 | 40002 |  |

RHD: Rheumatic heart diseases

**Supplementary** **Table** **2** Missing data proportions

| Characteristic | Missing number | Missing proportion |
| --- | --- | --- |
| Age | 0 | 0 |
| Sex | 0 | 0 |
| Race | 1129 | 0.41 |
| Education | 3184 | 1.16 |
| Income | 40261 | 14.72 |
| BMI | 1024 | 0.37 |
| Physical activity | 62376 | 22.80 |
| Smoke | 1333 | 0.49 |
| Alcohol | 641 | 0.23 |
| DASH | 20484 | 7.49 |
| History of diabetes mellitus | 0 | 0 |
| History of hypertension | 0 | 0 |
| History of CVD | 0 | 0 |
| History of cancer | 0 | 0 |

**Supplementary** **Table** **3** Baseline characteristics before and after imputation

| Characteristic | level | Incomplete data set without imputation(n=170343) | Incomplete data set with imputation(n=273595) |
| --- | --- | --- | --- |
| Age(mean(SD)) |  | 55.90 (8.09) | 56.56 (8.08) |
|  | <65 years | 142120 (83.43) | 221287 (80.88) |
|  | ≥65 years | 28223 (16.57) | 52308 (19.12) |
| Sex(%) | Female | 85798 (50.37) | 147678 (53.98) |
|  | Male | 84545 (49.63) | 125917 (46.02) |
| Race(%) | Other | 6463 ( 3.79) | 13584 ( 4.97) |
|  | White | 163880 (96.21) | 260011 (95.03) |
| Education(%) | No  university degree | 106723 (62.65) | 186114 (68.03) |
|  | University degree | 63620 (37.35) | 87481 (31.97) |
| Income(%) | <£18,000 | 34403 (20.20) | 65492 (23.94) |
|  | >£18,000 | 135940 (79.80) | 208103 (76.06) |
| BMI(mean(SD)) |  | 27.28 (4.64) | 27.46 (4.78) |
| Physical activity (%) | Low | 31625 (18.57) | 51353 (18.77) |
|  | Moderate | 69426 (40.76) | 109618 (40.07) |
|  | High | 69292 (40.68) | 112624 (41.16) |
| Smoke(%) | Never | 93096 (54.65) | 149478 (54.63) |
|  | Previous | 60139 (35.30) | 95173 (34.79) |
|  | Current | 17108 (10.04) | 28944 (10.58) |
| Alcohol(%) | Never | 5455 ( 3.20) | 11917 ( 4.36) |
|  | Previous | 5195 ( 3.05) | 9780 ( 3.57) |
|  | Current | 159693 (93.75) | 251898 (92.07) |
| DASH(mean(SD)) |  | 4.92 (1.34) | 4.90 (1.34) |
| History of diabetes mellitus (%) | No | 162007 (95.11) | 258506 (94.48) |
|  | Yes | 8336 ( 4.89) | 15089 ( 5.52) |
| History of hypertension (%) | No | 120392 (70.68) | 188037 (68.73) |
|  | Yes | 49951 (29.32) | 85558 (31.27) |
| History of CVD (%) | No | 157848 (92.66) | 251163 (91.80) |
|  | Yes | 12495 ( 7.34) | 22432 ( 8.20) |
| History of cancer (%) | No | 155619 (91.36) | 248655 (90.88) |
|  | Yes | 14724 ( 8.64) | 24940 ( 9.12) |

**Supplementary** **Table 4** Baseline characteristics in RHD and control group

| Characteristic | level | Overall  (n=273595) | Control  (n=267544) | RHD  (n=6051) | P |
| --- | --- | --- | --- | --- | --- |
| Age(mean(SD)) |  | 56.562(8.082) | 56.440(8.078) | 61.964(6.181) | <0.001 |
|  | <65 years | 221287(80.88) | 217784(81.40) | 3503(57.89) | <0.001 |
|  | ≥65 years | 52308(19.12) | 49760(18.60) | 2548(42.11) |  |
| Sex(%) | Female | 147678(53.98) | 145041(54.21) | 2637(43.58) | <0.001 |
|  | Male | 125917(46.02) | 122503(45.79) | 3414(56.42) |  |
| Race(%) | Other | 13584(4.97) | 13319(4.98) | 265(4.38) | 0.037 |
|  | White | 260011(95.03) | 254225(95.02) | 5786(95.62) |  |
| Education(%) | No  university degree | 186114(68.03) | 181449(67.82) | 4665(77.09) | <0.001 |
|  | University degree | 87481(31.97) | 86095(32.18) | 1386(22.91) |  |
| Income(%) | <£18,000 | 65492(23.94) | 63306(23.66) | 2186(36.13) | <0.001 |
|  | >£18,000 | 208103(76.06) | 204238(76.34) | 3865(63.87) |  |
| BMI (mean (SD)) |  | 27.456(4.784) | 27.428(4.765) | 28.687(5.400) | <0.001 |
| Physical activity (%) | Low | 51353(18.77) | 50066(18.71) | 1287(21.27) | <0.001 |
|  | Moderate | 109618(40.07) | 107290(40.10) | 2328(38.47) |  |
|  | High | 112624(41.16) | 110188(41.19) | 2436(40.26) |  |
| Smoke(%) | Never | 149478(54.63) | 146679(54.82) | 2799(46.26) | <0.001 |
|  | Previous | 95173(34.79) | 92645(34.63) | 2528(41.78) |  |
|  | Current | 28944(10.58) | 28220(10.55) | 724(11.96) |  |
| Alcohol(%) | Never | 11917(4.36) | 11594(4.33) | 323(5.34) | <0.001 |
|  | Previous | 9780(3.57) | 9466(3.54) | 314(5.19) |  |
|  | Current | 251898(92.07) | 246484(92.13) | 5414(89.47) |  |
| DASH(mean(SD)) |  | 4.898(1.342) | 4.899(1.341) | 4.847(1.363) | 0.003 |
| History of diabetes mellitus (%) | No | 258506(94.48) | 253222(94.65) | 5284(87.32) | <0.001 |
|  | Yes | 15089(5.52) | 14322(5.35) | 767(12.68) |  |
| History of hypertension (%) | No | 188037(68.73) | 185433(69.31) | 2604(43.03) | <0.001 |
|  | Yes | 85558(31.27) | 82111(30.69) | 3447(56.97) |  |
| History of CVD (%) | No | 251163(91.80) | 246749(92.23) | 4414(72.95) | <0.001 |
|  | Yes | 22432(8.20) | 20795(7.77) | 1637(27.05) |  |
| History of cancer (%) | No | 248655(90.88) | 243313(90.94) | 5342(88.28) | <0.001 |
|  | Yes | 24940(9.12) | 24231(9.06) | 709(11.72) |  |

BMI: Body mass index; DASH: Dietary approaches to stop hypertension; CVD: Cardiovascular disease; RHD: Rheumatic heart diseases.

**Supplementary** **Table** **5** Cox proportional hazards hypothesis test of BCAAs (quartile grouping variables) and chronic RHD

| Variables | BCAAs | | | Leucine | | | Isoleucine | | | Valine | | |
| --- | --- | --- | --- | --- | --- | --- | --- | --- | --- | --- | --- | --- |
|  | Chisq | df | P | Chisq | df | P | Chisq | df | P | Chisq | df | P |
| Exposure | 7.4670 | 3 | 0.0584 | 2.8377 | 3 | 0.4173 | 1.9709 | 3 | 0.5785 | 4.6575 | 3 | 0.1987 |
| Age | 2.7749 | 1 | 0.0958 | 2.7872 | 1 | 0.0950 | 2.7785 | 1 | 0.0955 | 2.7744 | 1 | 0.0958 |
| Sex | 3.0602 | 1 | 0.0802 | 3.0543 | 1 | 0.0805 | 3.0436 | 1 | 0.0811 | 3.0671 | 1 | 0.0799 |
| Race | 0.0124 | 1 | 0.9113 | 0.0116 | 1 | 0.9144 | 0.0129 | 1 | 0.9096 | 0.0124 | 1 | 0.9112 |
| Education | 0.2725 | 1 | 0.6016 | 0.2751 | 1 | 0.5999 | 0.2703 | 1 | 0.6031 | 0.2706 | 1 | 0.6029 |
| Income | 3.2343 | 1 | 0.0721 | 3.2438 | 1 | 0.0717 | 3.2330 | 1 | 0.0722 | 3.2243 | 1 | 0.0726 |
| Physical activity | 3.0043 | 2 | 0.2226 | 2.9925 | 2 | 0.2240 | 2.9930 | 2 | 0.2239 | 2.9971 | 2 | 0.2235 |
| Smoke | 0.2691 | 2 | 0.8741 | 0.2747 | 2 | 0.8717 | 0.2678 | 2 | 0.8747 | 0.2658 | 2 | 0.8756 |
| Alcohol | 1.5174 | 2 | 0.4683 | 1.5096 | 2 | 0.4701 | 1.5224 | 2 | 0.4671 | 1.5198 | 2 | 0.4677 |
| DASH | 1.6118 | 1 | 0.2042 | 1.6064 | 1 | 0.2050 | 1.6093 | 1 | 0.2046 | 1.6115 | 1 | 0.2043 |
| BMI | 0.0738 | 1 | 0.7859 | 0.0750 | 1 | 0.7842 | 0.0748 | 1 | 0.7845 | 0.0736 | 1 | 0.7861 |
| History of diabetes mellitus | 2.5068 | 1 | 0.1134 | 2.4971 | 1 | 0.1141 | 2.5051 | 1 | 0.1135 | 2.5092 | 1 | 0.1132 |
| History of hypertension | 3.6354 | 1 | 0.0566 | 3.6341 | 1 | 0.0566 | 3.6500 | 1 | 0.0561 | 3.6394 | 1 | 0.0564 |
| History of CVD | 3.6586 | 1 | 0.0558 | 3.6664 | 1 | 0.0555 | 3.6437 | 1 | 0.0563 | 3.6554 | 1 | 0.0559 |
| History of cancer | 1.7431 | 1 | 0.1868 | 1.7512 | 1 | 0.1857 | 1.7410 | 1 | 0.1870 | 1.7360 | 1 | 0.1876 |
| GLOBAL | 30.5155 | 20 | 0.0619 | 30.1112 | 20 | 0.0681 | 30.5814 | 20 | 0.0610 | 30.4748 | 20 | 0.0625 |

**Supplementary** **Table** **6** Cox proportional hazards hypothesis test of BCAAs (continuous variable) and chronic RHD

| Variables | BCAAs | | | Leucine | | | Isoleucine | | | Valine | | |
| --- | --- | --- | --- | --- | --- | --- | --- | --- | --- | --- | --- | --- |
|  | Chisq | df | P | Chisq | df | P | Chisq | df | P | Chisq | df | P |
| Exposure | 0.7580 | 1 | 0.3839 | 0.7174 | 1 | 0.3970 | 0.0129 | 1 | 0.9095 | 1.4979 | 1 | 0.2210 |
| Age | 2.7756 | 1 | 0.0957 | 2.7808 | 1 | 0.0954 | 2.7797 | 1 | 0.0955 | 2.7703 | 1 | 0.0960 |
| Sex | 3.0391 | 1 | 0.0813 | 3.0296 | 1 | 0.0818 | 3.0515 | 1 | 0.0807 | 3.0453 | 1 | 0.0810 |
| Race | 0.0118 | 1 | 0.9134 | 0.0115 | 1 | 0.9144 | 0.0125 | 1 | 0.9110 | 0.0121 | 1 | 0.9126 |
| Education | 0.2723 | 1 | 0.6018 | 0.2752 | 1 | 0.5998 | 0.2708 | 1 | 0.6028 | 0.2703 | 1 | 0.6031 |
| Income | 3.2471 | 1 | 0.0715 | 3.2623 | 1 | 0.0709 | 3.2430 | 1 | 0.0717 | 3.2367 | 1 | 0.0720 |
| Physical activity | 2.9932 | 2 | 0.2239 | 2.9894 | 2 | 0.2243 | 2.9928 | 2 | 0.2239 | 2.9955 | 2 | 0.2236 |
| Smoke | 0.2700 | 2 | 0.8737 | 0.2736 | 2 | 0.8722 | 0.2701 | 2 | 0.8737 | 0.2672 | 2 | 0.8749 |
| Alcohol | 1.5217 | 2 | 0.4673 | 1.5194 | 2 | 0.4678 | 1.5221 | 2 | 0.4672 | 1.5236 | 2 | 0.4668 |
| DASH | 1.6152 | 1 | 0.2038 | 1.6170 | 1 | 0.2035 | 1.6150 | 1 | 0.2038 | 1.6142 | 1 | 0.2039 |
| BMI | 0.0758 | 1 | 0.7831 | 0.0757 | 1 | 0.7832 | 0.0741 | 1 | 0.7855 | 0.0760 | 1 | 0.7829 |
| History of diabetes mellitus | 2.4977 | 1 | 0.1140 | 2.4915 | 1 | 0.1145 | 2.5088 | 1 | 0.1132 | 2.4981 | 1 | 0.1140 |
| History of hypertension | 3.6347 | 1 | 0.0566 | 3.6336 | 1 | 0.0566 | 3.6501 | 1 | 0.0561 | 3.6323 | 1 | 0.0567 |
| History of CVD | 3.6656 | 1 | 0.0555 | 3.6776 | 1 | 0.0551 | 3.6634 | 1 | 0.0556 | 3.6524 | 1 | 0.0560 |
| History of cancer | 1.7433 | 1 | 0.1867 | 1.7537 | 1 | 0.1854 | 1.7436 | 1 | 0.1867 | 1.7373 | 1 | 0.1875 |
| GLOBAL | 28.6084 | 18 | 0.0534 | 28.6464 | 18 | 0.0529 | 28.6505 | 18 | 0.0528 | 28.6576 | 18 | 0.0527 |

**Supplementary** **Table** **7** Mediation of immunity indicators in association between BCAAs and RHD

| Mediator | Effect | HR(95%CI) | *P* |
| --- | --- | --- | --- |
| AAPR | Direct effect | 1.071(1.034,1.077) | <0.001 |
|  | Indirect effect | 1.014(1.012,1.016) | <0.001 |
|  | Total effect | 1.086(1.05,1.094) | <0.001 |
|  | Mediation proportion (%) | 17.45(17.47,32.58) | <0.001 |
| LMR | Direct effect | 1.07(1.034,1.075) | <0.001 |
|  | Indirect effect | 1.008(1.005,1.009) | <0.001 |
|  | Total effect | 1.079(1.041,1.084) | <0.001 |
|  | Mediation proportion (%) | 11.07(7.99,17.68) | <0.001 |
| LCR | Direct effect | 1.058(1.022,1.061) | <0.001 |
|  | Indirect effect | 1.024(1.023,1.028) | <0.001 |
|  | Total effect | 1.083(1.045,1.091) | <0.001 |
|  | Mediation proportion (%) | 30.8(32.53,52.87) | <0.001 |
| PLR | Direct effect | 1.07(1.037,1.078) | <0.001 |
|  | Indirect effect | 1.006(1.002,1.007) | <0.001 |
|  | Total effect | 1.076(1.039,1.082) | <0.001 |
|  | Mediation proportion (%) | 7.92(4.89,12.12) | <0.001 |

Models were adjusted for age, sex, race, education, income, physical activity, smoke, alcohol, DASH, BMI, and the history of diabetes mellitus, hypertension, CVD, and cancer. BCAA: Branched-chain amino acid; BMI: Body mass index; DASH: Dietary approaches to stop hypertension; CVD: Cardiovascular disease; RHD: Rheumatic heart diseases; AAPR: Albumin to alkaline phosphatase ratio; LMR: Lymphocyte-to-monocyte ratio; LCR: Lymphocyte and C-reactive protein Ratio; PLR: Platelet-to-Lymphocyte Ratio.

**Supplementary** **Table 8** Mediation of immunity indicators in association between Isoleucine and RHD

| Mediator | Effect | HR(95%CI) | *P* |
| --- | --- | --- | --- |
| AAPR | Direct effect | 1.03(0.994,1.059) | <0.001 |
|  | Indirect effect | 1.016(1.014,1.018) | <0.001 |
|  | Total effect | 1.046(1.012,1.077) | <0.001 |
|  | Mediation proportion (%) | 35.3(21.43,87.64) | <0.001 |
| LMR | Direct effect | 1.035(1.001,1.064) | <0.001 |
|  | Indirect effect | 1.005(1.003,1.005) | <0.001 |
|  | Total effect | 1.04(1.006,1.069) | <0.001 |
|  | Mediation proportion (%) | 13.64(5.11,47.43) | <0.001 |
| LCR | Direct effect | 1.026(0.992,1.054) | <0.001 |
|  | Indirect effect | 1.017(1.017,1.019) | <0.001 |
|  | Total effect | 1.043(1.008,1.074) | <0.001 |
|  | Mediation proportion (%) | 39.33(18.01,68.90) | <0.001 |
| PLR | Direct effect | 1.033(1.002,1.064) | <0.001 |
|  | Indirect effect | 1.006(1.002,1.007) | <0.001 |
|  | Total effect | 1.038(1.004,1.069) | <0.001 |
|  | Mediation proportion (%) | 15.07(6.84,99.86) | <0.001 |

Models were adjusted for age, sex, race, education, income, physical activity, smoke, alcohol, DASH, BMI, and the history of diabetes mellitus, hypertension, CVD, and cancer. BMI: Body mass index; DASH: Dietary approaches to stop hypertension; CVD: Cardiovascular disease; RHD: Rheumatic heart diseases; AAPR: Albumin to alkaline phosphatase ratio; LMR: Lymphocyte-to-monocyte ratio; LCR: Lymphocyte and C-reactive protein Ratio; PLR: Platelet-to-Lymphocyte Ratio.

**Supplementary** **Table** **9** Mediation of immunity indicators in association between Valine and RHD

| Mediator | Effect | HR(95%CI) | *P* |
| --- | --- | --- | --- |
| AAPR | Direct effect | 1.072(1.042,1.119) | <0.001 |
|  | Indirect effect | 1.016(1.014,1.018) | <0.001 |
|  | Total effect | 1.09(1.061,1.139) | <0.001 |
|  | Mediation proportion (%) | 19.42(14.36,30.69) | <0.001 |
| LMR | Direct effect | 1.075(1.046,1.121) | <0.001 |
|  | Indirect effect | 1.007(1.004,1.007) | <0.001 |
|  | Total effect | 1.082(1.052,1.129) | <0.001 |
|  | Mediation proportion (%) | 8.55(4.42,11.7) | <0.001 |
| LCR | Direct effect | 1.058(1.03,1.101) | <0.001 |
|  | Indirect effect | 1.028(1.026,1.032) | <0.001 |
|  | Total effect | 1.087(1.057,1.136) | <0.001 |
|  | Mediation proportion (%) | 33.56(25.9,47.68) | <0.001 |
| PLR | Direct effect | 1.074(1.048,1.123) | <0.001 |
|  | Indirect effect | 1.006(1.002,1.007) | <0.001 |
|  | Total effect | 1.08(1.05,1.127) | <0.001 |
|  | Mediation proportion (%) | 7.55(3.58,8.77) | <0.001 |

Models were adjusted for age, sex, race, education, income, physical activity, smoke, alcohol, DASH, BMI, and the history of diabetes mellitus, hypertension, CVD, and cancer. BMI: Body mass index; DASH: Dietary approaches to stop hypertension; CVD: Cardiovascular disease; RHD: Rheumatic heart diseases; AAPR: Albumin to alkaline phosphatase ratio; LMR: Lymphocyte-to-monocyte ratio; LCR: Lymphocyte and C-reactive protein Ratio; PLR: Platelet-to-Lymphocyte Ratio.

**Supplementary** **Table 10** Sensitivity analysis of association between BCAAs and chronic RHD using accelerated failure time model

| Exposure | Crude Model | | Model 1 | | Model 2 | | Model 3 | |
| --- | --- | --- | --- | --- | --- | --- | --- | --- |
|  | HR(95%CI) | P | HR(95%CI) | P | HR(95%CI) | P | HR(95%CI) | P |
| BCAAs per 1SD | 1.025(1.012,1.038) | <0.001 | 1.026(1.013,1.038) | <0.001 | 1.024(1.011,1.037) | <0.001 | 1.024(1.011,1.037) | <0.001 |
| Q1 | ref |  | ref |  | ref |  | ref |  |
| Q2 | 1.04(1.002,1.079) | 0.04 | 1.029(0.992,1.068) | 0.128 | 1.029(0.992,1.068) | 0.128 | 1.029(0.992,1.068) | 0.124 |
| Q3 | 1.043(1.005,1.082) | 0.026 | 1.036(0.998,1.075) | 0.062 | 1.035(0.997,1.074) | 0.072 | 1.035(0.997,1.074) | 0.068 |
| Q4 | 1.085(1.047,1.125) | <0.001 | 1.086(1.047,1.126) | <0.001 | 1.081(1.042,1.121) | <0.001 | 1.081(1.043,1.121) | <0.001 |
| P for trend |  | <0.001 |  | <0.001 |  | <0.001 |  | <0.001 |
| Leucine per 1SD | 1.012(0.999,1.025) | 0.066 | 1.016(1.003,1.029) | 0.012 | 1.015(1.002,1.028) | 0.024 | 1.015(1.002,1.028) | 0.02 |
| Q1 | ref |  | ref |  | ref |  | ref |  |
| Q2 | 0.997(0.961,1.034) | 0.868 | 0.995(0.96,1.032) | 0.8 | 0.994(0.959,1.031) | 0.758 | 0.995(0.959,1.032) | 0.774 |
| Q3 | 1.011(0.975,1.048) | 0.564 | 1.015(0.979,1.053) | 0.408 | 1.014(0.978,1.051) | 0.458 | 1.015(0.978,1.052) | 0.434 |
| Q4 | 1.028(0.991,1.066) | 0.136 | 1.039(1.002,1.077) | 0.038 | 1.034(0.998,1.072) | 0.066 | 1.035(0.999,1.074) | 0.058 |
| P for trend |  | 0.098 |  | 0.02 |  | 0.038 |  | 0.032 |
| Isoleucine per 1SD | 1.022(1.009,1.034) | <0.001 | 1.021(1.009,1.034) | <0.001 | 1.019(1.007,1.032) | 0.002 | 1.02(1.007,1.032) | 0.002 |
| Q1 | ref |  | ref |  | ref |  | ref |  |
| Q2 | 1.018(0.981,1.057) | 0.34 | 1.01(0.973,1.048) | 0.594 | 1.007(0.971,1.045) | 0.696 | 1.008(0.971,1.046) | 0.684 |
| Q3 | 1.065(1.027,1.105) | <0.001 | 1.062(1.024,1.102) | 0.002 | 1.057(1.019,1.096) | 0.002 | 1.057(1.02,1.097) | 0.002 |
| Q4 | 1.058(1.02,1.097) | 0.002 | 1.057(1.019,1.096) | 0.002 | 1.05(1.013,1.089) | 0.008 | 1.051(1.013,1.09) | 0.008 |
| P for trend |  | <0.001 |  | <0.001 |  | <0.001 |  | <0.001 |
| Valine per 1SD | 1.033(1.02,1.046) | <0.001 | 1.032(1.019,1.045) | <0.001 | 1.03(1.017,1.043) | <0.001 | 1.031(1.018,1.044) | <0.001 |
| Q1 | ref |  | ref |  | ref |  | ref |  |
| Q2 | 1.033(0.995,1.072) | 0.092 | 1.018(0.981,1.057) | 0.348 | 1.02(0.982,1.058) | 0.304 | 1.02(0.983,1.059) | 0.298 |
| Q3 | 1.051(1.013,1.091) | 0.008 | 1.035(0.998,1.074) | 0.066 | 1.036(0.998,1.075) | 0.062 | 1.036(0.999,1.075) | 0.058 |
| Q4 | 1.102(1.062,1.142) | <0.001 | 1.095(1.056,1.135) | <0.001 | 1.09(1.051,1.131) | <0.001 | 1.091(1.052,1.131) | <0.001 |
| P for trend |  | <0.001 |  | <0.001 |  | <0.001 |  | <0.001 |

Model 1 adjusted for age, sex, race, education and income

Model 2	adjusted for Model 1+ physical activity, smoke, alcohol, and DASH

Model 3 adjusted for Model 2+ BMI, and the history of diabetes mellitus, hypertension, CVD, and cancer.

BCAA: Branched-chain amino acid; BMI: Body mass index; DASH: Dietary approaches to stop hypertension; CVD: Cardiovascular disease; RHD: Rheumatic heart diseases.

**Supplementary** **Table 11** Sensitivity analysis of association between BCAAs and chronic RHD using Cox Regression with age scale

| Exposure | Crude Model | | Model 1 | | Model 2 | | Model 3 | |
| --- | --- | --- | --- | --- | --- | --- | --- | --- |
|  | HR(95%CI) | P | HR(95%CI) | P | HR(95%CI) | P | HR(95%CI) | P |
| BCAAs per 1SD | 1.049(1.024,1.075) | <0.001 | 1.055(1.029,1.081) | <0.0001 | 1.048(1.022,1.074) | <0.001 | 1.048(1.023,1.074) | <0.001 |
| Q1 | ref |  | ref |  | ref |  | ref |  |
| Q2 | 1.080(1.004,1.161) | 0.040 | 1.065(0.990,1.145) | 0.092 | 1.058(0.984,1.139) | 0.127 | 1.047(0.973,1.126) | 0.218 |
| Q3 | 1.086(1.010,1.168) | 0.026 | 1.082(1.006,1.164) | 0.034 | 1.069(0.994,1.150) | 0.073 | 1.035(0.962,1.113) | 0.360 |
| Q4 | 1.174(1.093,1.261) | <0.0001 | 1.189(1.107,1.277) | <0.0001 | 1.165(1.084,1.251) | <0.0001 | 1.083(1.007,1.164) | 0.032 |
| P for trend |  | <0.0001 |  | <0.0001 |  | <0.0001 |  | 0.053 |
| Leucine per 1SD | 1.023(0.998,1.049) | 0.067 | 1.036(1.011,1.062) | 0.005 | 1.029(1.004,1.055) | 0.024 | 1.030(1.004,1.056) | 0.021 |
| Q1 | ref |  | ref |  | ref |  | ref |  |
| Q2 | 0.994(0.925,1.068) | 0.866 | 0.996(0.927,1.071) | 0.917 | 0.989(0.920,1.063) | 0.756 | 0.979(0.911,1.052) | 0.566 |
| Q3 | 1.021(0.951,1.097) | 0.566 | 1.040(0.968,1.117) | 0.281 | 1.027(0.956,1.104) | 0.460 | 1.000(0.930,1.074) | 0.995 |
| Q4 | 1.055(0.983,1.133) | 0.138 | 1.091(1.017,1.171) | 0.016 | 1.069(0.995,1.148) | 0.067 | 1.002(0.933,1.077) | 0.950 |
| P for trend |  | 0.099 |  | 0.008 |  | 0.038 |  | 0.807 |
| Isoleucine per 1SD | 1.043(1.018,1.069) | <0.001 | 1.045(1.020,1.071) | <0.001 | 1.039(1.013,1.064) | 0.003 | 1.039(1.014,1.065) | 0.002 |
| Q1 | ref |  | ref |  | ref |  | ref |  |
| Q2 | 1.036(0.963,1.115) | 0.339 | 1.022(0.950,1.099) | 0.565 | 1.015(0.943,1.092) | 0.697 | 1.005(0.934,1.081) | 0.904 |
| Q3 | 1.132(1.054,1.216) | <0.001 | 1.131(1.053,1.215) | <0.001 | 1.115(1.038,1.198) | 0.003 | 1.085(1.009,1.165) | 0.027 |
| Q4 | 1.117(1.040,1.200) | 0.003 | 1.123(1.045,1.206) | 0.002 | 1.101(1.025,1.183) | 0.009 | 1.037(0.964,1.115) | 0.327 |
| P for trend |  | <0.001 |  | <0.001 |  | <0.001 |  | 0.117 |
| Valine per 1SD | 1.065(1.040,1.092) | <0.0001 | 1.068(1.042,1.095) | <0.0001 | 1.061(1.035,1.087) | <0.0001 | 1.061(1.035,1.088) | <0.0001 |
| Q1 | ref |  | ref |  | ref |  | ref |  |
| Q2 | 1.065(0.990,1.146) | 0.092 | 1.042(0.969,1.122) | 0.267 | 1.039(0.965,1.118) | 0.307 | 1.028(0.955,1.107) | 0.458 |
| Q3 | 1.103(1.026,1.186) | 0.008 | 1.081(1.005,1.163) | 0.036 | 1.072(0.996,1.153) | 0.063 | 1.037(0.964,1.115) | 0.334 |
| Q4 | 1.209(1.126,1.299) | <0.0001 | 1.208(1.125,1.297) | <0.0001 | 1.186(1.104,1.274) | <0.0001 | 1.099(1.022,1.181) | 0.011 |
| P for trend |  | <0.0001 |  | <0.0001 |  | <0.0001 |  | 0.012 |

Model 1 adjusted for sex, race, education and income

Model 2	adjusted for Model 1+ physical activity, smoke, alcohol, and DASH

Model 3 adjusted for Model 2+ BMI, and the history of diabetes mellitus, hypertension, CVD, and cancer.

BCAA: Branched-chain amino acid; BMI: Body mass index; DASH: Dietary approaches to stop hypertension; CVD: Cardiovascular disease; RHD: Rheumatic heart diseases.

**Supplementary** **Table** **12** Sensitivity analysis of association between BCAAs and chronic RHD after excluding the participants with missing covariates

| Exposure | Crude Model | | Model 1 | | Model 2 | | Model 3 | |  |
| --- | --- | --- | --- | --- | --- | --- | --- | --- | --- |
|  | HR(95%CI) | P | HR(95%CI) | P | HR(95%CI) | P | HR(95%CI) | P |  |
| BCAAs per 1SD | 1.044(1.010,1.079) | 0.010 | 1.050(1.016,1.086) | 0.004 | 1.041(1.007,1.076) | 0.017 | 1.042(1.008,1.077) | 0.015 |  |
| Q1 | ref |  | ref |  | ref |  | ref |  |  |
| Q2 | 1.017(0.921,1.124) | 0.734 | 1.001(0.907,1.106) | 0.978 | 0.994(0.900,1.098) | 0.902 | 0.994(0.900,1.098) | 0.912 |  |
| Q3 | 1.073(0.973,1.183) | 0.159 | 1.071(0.971,1.181) | 0.170 | 1.056(0.957,1.164) | 0.278 | 1.058(0.959,1.167) | 0.258 |  |
| Q4 | 1.127(1.024,1.241) | 0.015 | 1.140(1.035,1.255) | 0.008 | 1.112(1.009,1.224) | 0.031 | 1.115(1.012,1.228) | 0.028 |  |
| P for trend |  | 0.007 |  | 0.003 |  | 0.014 |  | 0.011 |  |
| Leucine per 1SD | 1.020(0.987,1.055) | 0.237 | 1.033(0.999,1.068) | 0.058 | 1.023(0.990,1.058) | 0.175 | 1.025(0.991,1.060) | 0.151 |  |
| Q1 | ref |  | ref |  | ref |  | ref |  |  |
| Q2 | 0.976(0.884,1.077) | 0.625 | 0.975(0.883,1.076) | 0.614 | 0.964(0.874,1.064) | 0.472 | 0.965(0.875,1.066) | 0.484 |  |
| Q3 | 1.037(0.942,1.143) | 0.457 | 1.056(0.958,1.163) | 0.273 | 1.039(0.943,1.145) | 0.435 | 1.042(0.946,1.148) | 0.403 |  |
| Q4 | 1.041(0.946,1.146) | 0.411 | 1.072(0.974,1.180) | 0.154 | 1.045(0.949,1.150) | 0.372 | 1.049(0.952,1.155) | 0.333 |  |
| P for trend |  | 0.235 |  | 0.06 |  | 0.178 |  | 0.153 |  |
| Isoleucine per 1SD | 1.036(1.003,1.071) | 0.032 | 1.039(1.006,1.074) | 0.021 | 1.031(0.998,1.066) | 0.069 | 1.032(0.998,1.066) | 0.063 |  |
| Q1 | ref |  | ref |  | ref |  | ref |  |  |
| Q2 | 1.016(0.920,1.122) | 0.754 | 1.001(0.906,1.105) | 0.990 | 0.992(0.899,1.096) | 0.877 | 0.993(0.899,1.096) | 0.882 |  |
| Q3 | 1.135(1.031,1.249) | 0.010 | 1.136(1.032,1.251) | 0.009 | 1.117(1.015,1.230) | 0.024 | 1.119(1.016,1.232) | 0.023 |  |
| Q4 | 1.074(0.974,1.183) | 0.151 | 1.078(0.979,1.188) | 0.128 | 1.052(0.955,1.160) | 0.305 | 1.054(0.957,1.162) | 0.286 |  |
| P for trend |  | 0.04 |  | 0.024 |  | 0.085 |  | 0.077 |  |
| Valine per 1SD | 1.060(1.026,1.096) | <0.001 | 1.064(1.029,1.100) | <0.001 | 1.055(1.020,1.090) | 0.002 | 1.055(1.021,1.091) | 0.002 |  |
| Q1 | ref |  | ref |  | ref |  | ref |  |  |
| Q2 | 1.051(0.951,1.162) | 0.330 | 1.030(0.932,1.138) | 0.565 | 1.026(0.929,1.134) | 0.611 | 1.028(0.930,1.136) | 0.594 |  |
| Q3 | 1.103(0.999,1.217) | 0.051 | 1.085(0.983,1.197) | 0.105 | 1.074(0.973,1.186) | 0.154 | 1.077(0.975,1.188) | 0.143 |  |
| Q4 | 1.187(1.078,1.307) | <0.001 | 1.186(1.077,1.306) | <0.001 | 1.160(1.053,1.278) | 0.003 | 1.162(1.055,1.280) | 0.002 |  |
| P for trend |  | <0.001 |  | <0.001 |  | 0.001 |  | 0.001 |  |

The sample was 170,343

Model 1 adjusted for age, sex, race, education and income

Model 2	adjusted for Model 1+ physical activity, smoke, alcohol, and DASH

Model 3 adjusted for Model 2+ BMI, and the history of diabetes mellitus, hypertension, CVD, and cancer.

BCAA: Branched-chain amino acid; BMI: Body mass index; DASH: Dietary approaches to stop hypertension; CVD: Cardiovascular disease; RHD: Rheumatic heart diseases.

**Supplementary** **Table** **13** Sensitivity analysis of association between BCAAs and chronic RHD after excluding the participants who developed RHD within 2 years of follow-up.

| Exposure | Crude Model | | Model 1 | | Model 2 | | Model 3 | |
| --- | --- | --- | --- | --- | --- | --- | --- | --- |
|  | HR(95%CI) | P | HR(95%CI) | P | HR(95%CI) | P | HR(95%CI) | P |
| BCAAs per 1SD | 1.051(1.025,1.078) | <0.0001 | 1.057(1.031,1.084) | <0.0001 | 1.050(1.024,1.077) | <0.001 | 1.051(1.024,1.077) | <0.001 |
| Q1 | ref |  | ref |  | ref |  | ref |  |
| Q2 | 1.101(1.022,1.186) | 0.011 | 1.086(1.008,1.170) | 0.030 | 1.079(1.002,1.163) | 0.045 | 1.068(0.991,1.150) | 0.083 |
| Q3 | 1.094(1.016,1.179) | 0.018 | 1.090(1.012,1.174) | 0.023 | 1.077(0.999,1.160) | 0.052 | 1.044(0.968,1.125) | 0.264 |
| Q4 | 1.187(1.103,1.276) | <0.0001 | 1.202(1.117,1.293) | <0.0001 | 1.177(1.094,1.267) | <0.0001 | 1.097(1.019,1.181) | 0.014 |
| P for trend |  | <0.0001 |  | <0.0001 |  | <0.0001 |  | 0.034 |
| Leucine per 1SD | 1.026(1.001,1.052) | 0.045 | 1.039(1.013,1.066) | 0.003 | 1.032(1.006,1.058) | 0.015 | 1.032(1.007,1.059) | 0.013 |
| Q1 | ref |  | ref |  | ref |  | ref |  |
| Q2 | 0.997(0.926,1.073) | 0.938 | 0.999(0.929,1.076) | 0.988 | 0.992(0.921,1.067) | 0.823 | 0.982(0.913,1.057) | 0.636 |
| Q3 | 1.028(0.955,1.106) | 0.463 | 1.047(0.973,1.126) | 0.218 | 1.034(0.961,1.112) | 0.373 | 1.007(0.936,1.084) | 0.853 |
| Q4 | 1.063(0.989,1.143) | 0.097 | 1.100(1.023,1.182) | 0.010 | 1.077(1.001,1.158) | 0.046 | 1.012(0.941,1.089) | 0.746 |
| P for trend |  | 0.066 |  | 0.004 |  | 0.024 |  | 0.604 |
| Isoleucine per 1SD | 1.043(1.018,1.070) | <0.001 | 1.046(1.020,1.072) | <0.001 | 1.039(1.014,1.065) | 0.003 | 1.040(1.014,1.066) | 0.002 |
| Q1 | ref |  | ref |  | ref |  | ref |  |
| Q2 | 1.041(0.966,1.121) | 0.294 | 1.026(0.952,1.106) | 0.496 | 1.019(0.946,1.098) | 0.620 | 1.009(0.937,1.087) | 0.810 |
| Q3 | 1.137(1.057,1.224) | <0.001 | 1.137(1.057,1.223) | <0.001 | 1.121(1.042,1.206) | 0.002 | 1.091(1.014,1.174) | 0.019 |
| Q4 | 1.120(1.041,1.205) | 0.002 | 1.126(1.047,1.212) | 0.001 | 1.104(1.026,1.188) | 0.008 | 1.042(0.968,1.122) | 0.277 |
| P for trend |  | <0.001 |  | <0.001 |  | <0.001 |  | 0.095 |
| Valine per 1SD | 1.068(1.042,1.095) | <0.0001 | 1.071(1.044,1.098) | <0.0001 | 1.063(1.037,1.090) | <0.0001 | 1.064(1.037,1.091) | <0.0001 |
| Q1 | ref |  | ref |  | ref |  | ref |  |
| Q2 | 1.077(1.000,1.161) | 0.051 | 1.055(0.978,1.137) | 0.165 | 1.051(0.975,1.133) | 0.195 | 1.040(0.965,1.122) | 0.301 |
| Q3 | 1.115(1.035,1.201) | 0.004 | 1.093(1.014,1.177) | 0.019 | 1.083(1.005,1.167) | 0.036 | 1.049(0.974,1.130) | 0.209 |
| Q4 | 1.219(1.134,1.311) | <0.0001 | 1.218(1.133,1.310) | <0.0001 | 1.196(1.111,1.286) | <0.0001 | 1.111(1.032,1.196) | 0.005 |
| P for trend |  | <0.0001 |  | <0.0001 |  | <0.0001 |  | 0.006 |

The sample was 273,368

Model 1 adjusted for age, sex, race, education and income

Model 2	adjusted for Model 1+ physical activity, smoke, alcohol, and DASH

Model 3 adjusted for Model 2+ BMI, and the history of diabetes mellitus, hypertension, CVD, and cancer.

BCAA: Branched-chain amino acid; BMI: Body mass index; DASH: Dietary approaches to stop hypertension; CVD: Cardiovascular disease; RHD: Rheumatic heart diseases.

**Supplementary** **Table** **14** Sensitivity analysis of association between BCAAs and chronic RHD after excluding the participants who has the history of CVD

| Exposure | Crude Model | | Model 1 | | Model 2 | | Model 3 | |
| --- | --- | --- | --- | --- | --- | --- | --- | --- |
|  | HR(95%CI) | P | HR(95%CI) | P | HR(95%CI) | P | HR(95%CI) | P |
| BCAAs per 1SD | 1.021(1.013,1.051) | 0.016 | 1.031(1.001,1.062) | 0.041 | 1.026(1.012,1.056) | 0.037 | 1.027(1.011,1.057) | 0.029 |
| Q1 | ref |  | ref |  | ref |  | ref |  |
| Q2 | 1.044(0.961,1.135) | 0.309 | 1.032(0.950,1.122) | 0.453 | 1.028(0.946,1.117) | 0.518 | 1.029(0.946,1.118) | 0.508 |
| Q3 | 1.028(0.946,1.118) | 0.514 | 1.033(0.950,1.124) | 0.448 | 1.024(0.942,1.114) | 0.577 | 1.026(0.943,1.116) | 0.555 |
| Q4 | 1.063(1.029,1.155) | 0.005 | 1.091(1.003,1.186) | 0.042 | 1.075(1.009,1.169) | 0.029 | 1.077(1.005,1.172) | 0.041 |
| P for trend |  | 0.214 |  | 0.053 |  | 0.113 |  | 0.103 |
| Leucine per 1SD | 0.992(0.963,1.022) | 0.610 | 1.010(0.980,1.040) | 0.517 | 1.004(0.975,1.034) | 0.785 | 1.005(0.976,1.035) | 0.736 |
| Q1 | ref |  | ref |  | ref |  | ref |  |
| Q2 | 0.970(0.893,1.053) | 0.465 | 0.975(0.898,1.059) | 0.545 | 0.968(0.891,1.051) | 0.437 | 0.969(0.892,1.052) | 0.453 |
| Q3 | 0.940(0.865,1.022) | 0.147 | 0.967(0.889,1.051) | 0.424 | 0.956(0.880,1.040) | 0.295 | 0.958(0.882,1.042) | 0.319 |
| Q4 | 0.967(0.891,1.051) | 0.434 | 1.015(0.934,1.102) | 0.730 | 0.998(0.918,1.085) | 0.964 | 1.001(0.921,1.088) | 0.986 |
| P for trend |  | 0.325 |  | 0.811 |  | 0.879 |  | 0.933 |
| Isoleucine per 1SD | 1.019(0.990,1.049) | 0.208 | 1.025(0.995,1.055) | 0.098 | 1.020(0.991,1.050) | 0.178 | 1.021(0.991,1.051) | 0.168 |
| Q1 | ref |  | ref |  | ref |  | ref |  |
| Q2 | 1.004(0.923,1.091) | 0.929 | 0.994(0.914,1.081) | 0.890 | 0.989(0.909,1.075) | 0.793 | 0.990(0.910,1.076) | 0.809 |
| Q3 | 1.044(0.961,1.135) | 0.306 | 1.053(0.969,1.145) | 0.222 | 1.043(0.959,1.133) | 0.324 | 1.044(0.960,1.134) | 0.313 |
| Q4 | 1.031(0.948,1.121) | 0.473 | 1.051(0.966,1.142) | 0.247 | 1.036(0.953,1.127) | 0.405 | 1.038(0.955,1.129) | 0.383 |
| P for trend |  | 0.329 |  | 0.127 |  | 0.237 |  | 0.222 |
| Valine per 1SD | 1.039(1.010,1.070) | 0.009 | 1.045(1.015,1.076) | 0.003 | 1.041(1.011,1.072) | 0.007 | 1.041(1.011,1.072) | 0.007 |
| Q1 | ref |  | ref |  | ref |  | ref |  |
| Q2 | 1.024(0.941,1.114) | 0.587 | 1.004(0.923,1.093) | 0.919 | 1.003(0.922,1.092) | 0.942 | 1.004(0.922,1.092) | 0.930 |
| Q3 | 1.063(0.977,1.156) | 0.155 | 1.050(0.966,1.142) | 0.251 | 1.046(0.961,1.137) | 0.298 | 1.047(0.962,1.139) | 0.287 |
| Q4 | 1.105(1.017,1.201) | 0.018 | 1.117(1.028,1.214) | 0.009 | 1.104(1.016,1.201) | 0.020 | 1.106(1.017,1.202) | 0.018 |
| P for trend |  | 0.012 |  | 0.005 |  | 0.012 |  | 0.011 |

The sample was 251,163

Model 1 adjusted for age, sex, race, education and income

Model 2	adjusted for Model 1+ physical activity, smoke, alcohol, and DASH

Model 3 adjusted for Model 2+ BMI, and the history of diabetes mellitus, hypertension, and cancer.

BCAA: Branched-chain amino acid; BMI: Body mass index; DASH: Dietary approaches to stop hypertension; CVD: Cardiovascular disease; RHD: Rheumatic heart diseases.

**Supplementary Fig.** 1 Flowchart of this study

BCAA: Branched-chain amino acid; RHD: Rheumatic heart diseases

**Supplementary Fig.** 2 Missing data patterns

**Supplementary Fig.** 3 Cox proportional hazards hypothesis test of BCAAs (quartile grouping variable) and chronic RHD

**Supplementary Fig.** 4 Cox proportional hazards hypothesis test of Leucine (quartile grouping variable) and chronic RHD

**Supplementary Fig.** 5 Cox proportional hazards hypothesis test of Isoleucine (quartile grouping variable) and chronic RHD

**Supplementary Fig.** 6 Cox proportional hazards hypothesis test of Valine (quartile grouping variable) and chronic RHD

**Supplementary Fig.** 7 Cox proportional hazards hypothesis test of BCAAs (continuous variable) and chronic RHD

**Supplementary Fig.** 8 Cox proportional hazards hypothesis test of Leucine (continuous variable) and chronic RHD

**Supplementary Fig.** 9 Cox proportional hazards hypothesis test of Isoleucine (continuous variable) and chronic RHD

**Supplementary Fig.** 10 Cox proportional hazards hypothesis test of Valine (continuous variable) and chronic RHD

**Supplementary Fig.** 11 Residual plot of Cox proportional hazards model diagnostic

**Supplementary Fig.** 12 Subgroup analysis of association between Leucine and chronic RHD

Models were adjusted for age, sex, race, education, income, physical activity, smoke, alcohol, DASH, BMI, and the history of diabetes mellitus, hypertension, CVD, and cancer. BCAA: Branched-chain amino acid; BMI: Body mass index; DASH: Dietary approaches to stop hypertension; CVD: Cardiovascular disease; RHD: Rheumatic heart diseases.

**Supplementary Fig.** 13 Subgroup analysis of association between Isoleucine and chronic RHD

Models were adjusted for age, sex, race, education, income, physical activity, smoke, alcohol, DASH, BMI, and the history of diabetes mellitus, hypertension, CVD, and cancer. BCAA: Branched-chain amino acid; BMI: Body mass index; DASH: Dietary approaches to stop hypertension; CVD: Cardiovascular disease; RHD: Rheumatic heart diseases.

**Supplementary Fig.** 14 Subgroup analysis of association between Valine and chronic RHD

Models were adjusted for age, sex, race, education, income, physical activity, smoke, alcohol, DASH, BMI, and the history of diabetes mellitus, hypertension, CVD, and cancer. BCAA: Branched-chain amino acid; BMI: Body mass index; DASH: Dietary approaches to stop hypertension; CVD: Cardiovascular disease; RHD: Rheumatic heart diseases.

**Supplementary Fig.** 15 Mediating proportion of immunity in the relationship between Isoleucine and RHD

Models were adjusted for age, sex, race, education, income, physical activity, smoke, alcohol, DASH, BMI, and the history of diabetes mellitus, hypertension, CVD, and cancer. BCAA: Branched-chain amino acid; BMI: Body mass index; DASH: Dietary approaches to stop hypertension; CVD: Cardiovascular disease; RHD: Rheumatic heart diseases; AAPR: Albumin to alkaline phosphatase ratio; LMR: Lymphocyte-to-monocyte ratio; LCR: Lymphocyte and C-reactive protein Ratio; PLR: Platelet-to-Lymphocyte Ratio.

**Supplementary Fig.** 16 Mediating proportion of immunity in the relationship between Valine and RHD

Models were adjusted for age, sex, race, education, income, physical activity, smoke, alcohol, DASH, BMI, and the history of diabetes mellitus, hypertension, CVD, and cancer. BCAA: Branched-chain amino acid; BMI: Body mass index; DASH: Dietary approaches to stop hypertension; CVD: Cardiovascular disease; RHD: Rheumatic heart diseases; AAPR: Albumin to alkaline phosphatase ratio; LMR: Lymphocyte-to-monocyte ratio; LCR: Lymphocyte and C-reactive protein Ratio; PLR: Platelet-to-Lymphocyte Ratio.

**Supplementary Fig.** 17 RCS analysis of association between BCAAs with RHD using Cox Regression with age scale

Models were adjusted for sex, race, education, income, physical activity, smoke, alcohol, DASH, BMI, and the history of diabetes mellitus, hypertension, CVD, and cancer. BCAA: Branched-chain amino acid; BMI: Body mass index; DASH: Dietary approaches to stop hypertension; CVD: Cardiovascular disease; RHD: Rheumatic heart diseases; RCS: Restricted cubic spline.

**Supplementary Fig.** 18 RCS analysis of association between BCAAs with RHD after excluding the participants with missing covariates

Models were adjusted for age, sex, race, education, income, physical activity, smoke, alcohol, DASH, BMI, and the history of diabetes mellitus, hypertension, CVD, and cancer. BCAA: Branched-chain amino acid; BMI: Body mass index; DASH: Dietary approaches to stop hypertension; CVD: Cardiovascular disease; RHD: Rheumatic heart diseases; RCS: Restricted cubic spline.

**Supplementary Fig.** 19 RCS analysis of association between BCAAs with RHD after excluding the participants who developed RHD within 2 years of follow-up.

Models were adjusted for age, sex, race, education, income, physical activity, smoke, alcohol, DASH, BMI, and the history of diabetes mellitus, hypertension, CVD, and cancer. BCAA: Branched-chain amino acid; BMI: Body mass index; DASH: Dietary approaches to stop hypertension; CVD: Cardiovascular disease; RHD: Rheumatic heart diseases; RCS: Restricted cubic spline.

**Supplementary Fig.** 20 RCS analysis of association between BCAAs with RHD after excluding the participants who has the history of CVD.

Models were adjusted for age, sex, race, education, income, physical activity, smoke, alcohol, DASH, BMI, and the history of diabetes mellitus, hypertension, and cancer. BCAA: Branched-chain amino acid; BMI: Body mass index; DASH: Dietary approaches to stop hypertension; CVD: Cardiovascular disease; RHD: Rheumatic heart diseases; RCS: Restricted cubic spline.
